# Surface Microstructural Responses of Heterogeneous Green Schist to Femtosecond Laser Grooving with Varying Process Parameters

**DOI:** 10.3390/ma18163751

**Published:** 2025-08-11

**Authors:** Chengaonan Wang, Kai Li, Xianshi Jia, Cong Wang, Yansong Wang, Zheng Yuan

**Affiliations:** 1 Wuhan University, Wuhan 430072, China; wcgn1818@126.com (C.W.); 00011853@whu.edu.cn (Y.W.); 2 State Key Laboratory of Precision Manufacturing for Extreme Service Performance, College of Mechanical and Electrical Engineering, Central South University, Changsha 410083, China; likai01@csu.edu.cn (K.L.); 221026@csu.edu.cn (X.J.); wangcong@csu.edu.cn (C.W.)

**Keywords:** femtosecond laser, green schist, Wudang Mountain, micro/nanostructures, water contact angle

## Abstract

The Mount Wudang architectural complex, recognized as a UNESCO World Cultural Heritage site, extensively utilizes green schist as the building material in its rock temple structures. Due to prolonged exposure to weathering and moisture, effective surface protection of these stones is crucial for their preservation. Inspired by the lotus leaf, femtosecond laser fabrication of bioinspired micro/nanostructures offers a promising approach for imparting hydrophobicity to stone surfaces. However, green schist is a typical heterogeneous material primarily composed of quartz, chlorite, and muscovite, and it contains metal elements, such as Fe and Ni. These pronounced compositional differences complicate laser–material interactions, posing considerable challenges to the formation of stable and uniform micro/nanostructures. To address this issue, we performed systematic femtosecond laser scanning experiments on green schist surfaces using a 100 kHz, 40 μJ laser with a 30 μm spot diameter, fabricating microgrooves under various process conditions. Surface morphology and EDS mapping analyses were conducted to elucidate the ablation responses of quartz, chlorite, and muscovite under different groove spacings (100 μm, 80 μm, 60 μm, and 40 μm) and scan repetitions (1, 2, 4, 6, 8, 10). The results revealed distinct differences in energy absorption, material ejection, and surface reorganization among these minerals, significantly influencing the formation mechanisms of laser-induced structures. Based on optimized parameters (60 μm spacing, 2–6 passes), robust and repeatable micro/nanostructures were successfully produced, yielding superhydrophobic performance with contact angles exceeding 155°. This work offers a novel strategy for interface control in heterogeneous natural stone materials and provides a theoretical and technical foundation for the protection and functional modification of green schist in heritage conservation.

## 1. Introduction

Mount Wudang, located in Hubei Province, China, is one of the most culturally and historically significant mountain ranges in East Asia. Renowned as the birthplace of Taoism and Wudang martial arts, it houses a complex of ancient palaces, temples, and shrines that were constructed over several dynastic periods, especially during the Ming dynasty. Recognized as a UNESCO World Heritage Site, Mount Wudang exemplifies the profound integration of natural landscapes with religious architecture, much of which was carved directly into native stone [1,2,3]. These stone structures, including cliffside shrines and rock-cut temples, reflect not only architectural ingenuity but also the Taoist philosophy of harmony between humans and nature. However, centuries of exposure to environmental stressors, particularly water infiltration, have resulted in progressive structural degradation, threatening both the material integrity and cultural value of these unique stone heritage sites. Preserving these relics thus demands advanced, non-invasive technologies tailored to the heterogeneous and fragile nature of their stone substrates [4,5,6].

Among various deterioration mechanisms, water ingress plays a dominant role in the weathering of exposed stone heritage. Water infiltrates through microcracks and pores, accelerating physical and chemical degradation processes, such as freeze–thaw cycles, salt crystallization, and mineral dissolution [7,8,9]. These effects are especially pronounced in green schist, a common lithology at Mount Wudang, characterized by its foliated texture and compositional heterogeneity. Green schist typically comprises quartz, chlorite, and muscovite, with significant variability in hardness, thermal conductivity, and optical absorption across mineral phases. Such complexity presents a major challenge for developing protective strategies that are both effective and non-destructive. A promising approach lies in modifying the stone surface to exhibit water-repellent properties without compromising the appearance or chemical composition of the substrate [10,11,12].

Inspired by natural superhydrophobic surfaces, most notably the lotus leaf, researchers have turned to biomimetic design for engineering hydrophobicity [13,14,15]. The lotus effect is attributed to a hierarchical surface structure combining micro- and nanoscale roughness with low surface energy, which allows water droplets to roll off, removing dust and minimizing surface wetting [16]. Reproducing this effect artificially has led to a new generation of functional surfaces that resist moisture penetration, improve durability, and require minimal maintenance [17,18]. When applied to stone heritage conservation, such biomimetic strategies offer a non-chemical, reversible means of protection that aligns with international principles of minimal intervention and material compatibility. In this context, laser-based micro-nanostructuring has emerged as a powerful method for fabricating lotus-inspired textures [19,20,21]. Compared to traditional coatings or chemical treatments, laser structuring, such as ultrafast laser processing or nanosecond laser processing, provides unmatched spatial precision and long-term stability. It enables the fabrication of complex surface morphologies that can modulate wettability, friction, or optical properties without introducing foreign materials or inducing chemical reactions [22,23,24]. Among various laser technologies, femtosecond lasers offer distinct advantages for delicate materials, such as heritage stone. The capability of “cold ablation” distinguishes femtosecond lasers from longer-pulse alternatives (e.g., nanosecond lasers) [25,26], which often produce thermal damage, melting, or cracking—undesirable outcomes for fragile, compositionally diverse stones [27,28]. In the context of heritage preservation, where even minor thermal effects can compromise structural integrity or aesthetic authenticity, the precision and non-thermal nature of femtosecond processing make it an ideal candidate for surface modification [29].

Despite its promise, femtosecond laser processing of natural stones, such as green schist, remains poorly understood due to their inherent heterogeneity and anisotropy. The distinct physical and optical properties of individual minerals lead to variable energy absorption, ablation thresholds, and material removal dynamics during laser exposure [30,31,32]. For instance, chlorite may respond differently from quartz under identical irradiation conditions, resulting in uneven surface topography or localized overprocessing. These phenomena necessitate a nuanced understanding of mineral-specific interactions and careful optimization of processing parameters to ensure consistent and functional surface patterning. In this regard, femtosecond laser-induced wettability modification has been well-demonstrated on compositionally homogeneous materials, such as high-purity marble. For example, López et al. [30] processed grooves and holes on calcite-rich, equigranular white marble and achieved a significant increase in the water contact angle (WCA) from 66° to 134°, attributing the hydrophobicity to stable micro/nano hierarchical structures and the Cassie–Baxter wetting regime. Similarly, Ariza et al. [16] fabricated lotus-inspired textures on marble using a 500 kHz femtosecond laser, and observed a gradual increase in WCA over time, eventually reaching approximately 140°, suggesting a dynamic change in surface properties post-laser treatment. 

In contrast, in the context of femtosecond laser processing and hydrophobic surface engineering of heterogeneous materials, López et al. [11] demonstrated that only compositionally uniform marble maintained consistent hydrophobic behavior after laser treatment, while materials, such as slate, quartzite, and granite, exhibited a lower WCA and irregular textures due to their intrinsic heterogeneity. More recent studies [12] have further investigated how non-homogeneous stone compositions respond to femtosecond laser ablation, highlighting the critical role of material uniformity in structure formation and functional performance. Among the most influential parameters in femtosecond laser microstructuring are groove spacing (pitch) and the number of scan passes [21,24,33]. Groove spacing controls the density and continuity of surface textures, thereby influencing surface roughness, droplet adhesion, and hydrophobic behavior. The number of laser passes determines the depth and clarity of the grooves, as well as the potential for re-melting, redeposition, or phase transformation [34]. In heterogeneous materials, like green schist, variations in these parameters can significantly affect the reproducibility and effectiveness of microstructure formation. Hence, a systematic exploration of how pitch and scan number affect the microstructural evolution of green schist surfaces under femtosecond irradiation is essential for developing robust and scalable processing strategies.

To date, limited studies have addressed how natural multi-phase rocks respond to controlled femtosecond laser patterning, particularly under varying texturing conditions. Understanding the formation mechanisms of micro/nanostructures, the mineral-specific ablation responses, and the overall surface functionality remains a crucial gap in both laser–material interaction research and cultural heritage engineering. This study addresses these challenges by systematically investigating the surface microstructural responses of heterogeneous green schist subjected to femtosecond laser grooving under different processing conditions. Specifically, we evaluate the influence of groove spacing and scan passes on ablation morphology, surface texture fidelity, and mineral-dependent behavior. By employing high-resolution imaging and analytical techniques, we elucidate the mechanisms governing structure formation and laser–matter interactions at both the micro- and nanoscale. The outcomes of this research not only deepen our understanding of ultrafast laser interaction with complex natural materials but also contribute to the development of non-invasive, functional surface treatments aimed at prolonging the life of historically significant stone heritage, such as that of Mount Wudang.

## 2. Materials and Methods

### 2.1. Materials 

**Figure 1:**
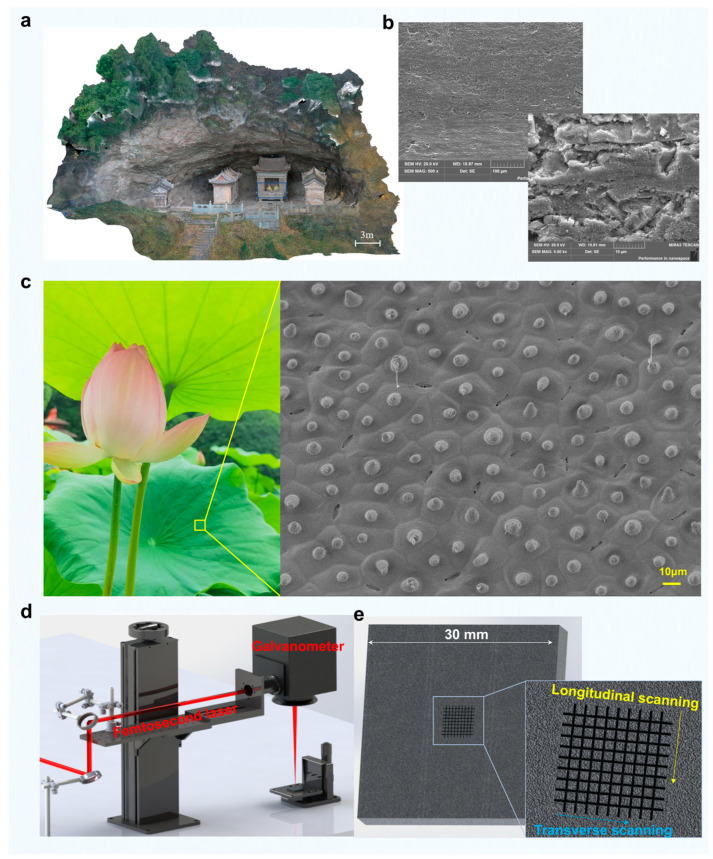
Bioinspired fabrication of micro/nanostructures on green schist surface based on lotus leaf microstructure features. (**a**,**b**) Characteristics of green schist in the construction stone of a rock temple in Mountain Wudang. (**c**) Characteristics of the micro/nanostructure features of a lotus leaf surface. (**d**,**e**) Femtosecond laser processing system and strategies for micro/nanostructure fabrication on the green schist surface.

In this study, green schist samples were collected from the Huagangyan area, a geologically representative site located within the broader Wudang Mountain region (Figure 1a). The selected specimens were derived from larger green schist blocks through precision cutting to obtain uniform samples measuring 30 mm × 30 mm × 5 mm. Two different cutting orientations were considered: one along the longitudinal direction (parallel to schistosity) and the other along the transverse direction (perpendicular to schistosity). Given the anisotropic nature of green schist, the influence of mineral alignment on microstructural evolution under femtosecond laser irradiation was preliminarily evaluated. Comparative analysis of laser-fabricated microstructures on longitudinal and transverse surfaces indicated more stable and uniform features on the latter. Consequently, transverse-cut samples were selected for all subsequent experiments to ensure reproducibility and minimize topographic irregularities induced by mineral foliation. To investigate the surface morphology of the native green schist, scanning electron microscopy (SEM) was employed prior to laser treatment. The SEM images revealed a relatively smooth topography at the microscale, but no clear distinction could be observed among the various mineral phases, such as chlorite, actinolite, or quartz, due to the resolution limits of conventional secondary electron imaging (Figure 1b). These findings suggest that optical or compositional contrast between mineral domains is not pronounced at the submicron scale without further elemental or crystallographic mapping.

In parallel, natural lotus leaves were used as a biological reference model to guide the design of artificial micro/nanostructures. Selected lotus leaves were carefully harvested and cleaned with deionized water and ethanol to remove contaminants, then air-dried under ambient conditions (Figure 1c). SEM imaging of the leaf surfaces revealed a hierarchical structure composed of dome-shaped protrusions with diameters in the range of 10–15 μm, superimposed by nanoscale wax crystals (Figure 1d). This complex surface architecture, known to facilitate superhydrophobicity through the Cassie–Baxter wetting regime, served as a bioinspiration for laser patterning strategies in this work. The observed micro-protrusions provided critical dimensional benchmarks for optimizing groove spacing and laser scan patterns on the green schist substrates. To replicate lotus leaf-like structures on green schist surfaces and achieve comparable hydrophobic performance for protective purposes, we employed a laser grooving method. The characteristic protrusion size of the lotus leaf (10–15 μm) served as a reference point in designing the laser scan parameters. By systematically varying the groove spacing and number of passes, and analyzing the resulting surface morphology and water contact angles, we were able to optimize the laser structuring strategy to approximate both the surface texture and hydrophobicity of the lotus leaf.

### 2.2. Experimental Setup

To fabricate hydrophobic micro- and nano-scale structures on green schist surfaces, femtosecond laser ablation was employed due to its high precision and minimal thermal damage. The laser system used in this study delivered ultrashort pulses with a pulse duration of 238 fs at a central wavelength of 1035 nm. The beam was focused onto the sample surface using an 80 mm focal-length telecentric lens, yielding a spot diameter of approximately 30 μm on the substrate. In order to minimize heat accumulation effects while maintaining reasonable processing efficiency, a laser repetition rate of 100 kHz was selected. Following preliminary parameter tuning, the pulse energy for microgroove fabrication was fixed at 40 μJ, and the scanning speed was set at 60 mm/s to ensure sufficient ablation depth while preserving the underlying mineral structure. The two-dimensional galvanometer is controlled by a Golden Orange control system (LMC2014, China) to achieve high-speed scanning.

To systematically investigate the influence of laser parameters on the resulting surface morphology and wettability, two sets of processing variables were selected: groove spacing (100 μm, 80 μm, 60 μm, and 40 μm) and number of scan passes (1, 2, 4, 6, 8, and 10). These parameters were combined to generate a comprehensive matrix of surface patterns with varied periodicity and groove depth. The wider groove spacing allowed for larger air-trapping pockets, while the increased number of passes enhanced the depth and sharpness of the microstructures. By varying these key parameters, a wide range of surface textures was generated, ranging from shallow, periodic microchannels to densely packed, hierarchically rough surfaces. This approach enabled a detailed evaluation of how both lateral and vertical aspects of the fabricated structures influence the hydrophobic transition on green schist, particularly in the context of mimicking the multiscale features observed on natural lotus leaves. For each set of grooving parameters, three repeated experiments were conducted.

### 2.3. Characterization of Surface Morphology and Wettability

To comprehensively evaluate the micro/nano-scale surface structures formed on green schist by femtosecond laser grooving, a combination of imaging, compositional, and wettability analyses was conducted. The three-dimensional (3D) topographies and surface height profiles of the processed areas were obtained using a laser confocal microscope (Olympus DSX1000, Tokyo, Japan). Before the subsequent sample analysis, the sample surfaces were coated with a thin layer of silver nanoparticles via spraying to enhance surface conductivity and reduce charging artifacts. Surface morphology was further characterized using scanning electron microscopy (SEM; MIRA3 LMU, TESCAN, Brno, Czech Republic) and backscattered electron imaging (BSE) to reveal compositional contrasts. Energy-dispersive X-ray spectroscopy (EDS), integrated into the SEM system, was employed to perform elemental analysis of different mineral domains.

As a first step, detailed analyses were conducted on samples processed under a selected set of laser parameters to identify the distinct surface responses of major mineral constituents, such as quartz, chlorite, and muscovite. These observations included comparative evaluation of ablation depth, and morphology development among the minerals. The corresponding surface topographies were documented using optical microscopy as well as 3D profilometry to correlate spatial geometry with mineral-specific behavior under laser irradiation. Subsequently, EDS mapping was used to investigate how the spatial distribution of elements was affected by laser structuring, enabling a more precise understanding of laser–matter interaction mechanisms in heterogeneous rock matrices. Finally, a broader set of samples processed with varying groove spacings and number of scan passes was systematically characterized to examine the evolution of micro/nanostructures and their corresponding compositional profiles. Water contact angle (WCA) measurements were performed using an optical tensiometer (OneAttension, Biolin Scientific, Espoo, Finland), equipped with a high-resolution CCD camera for real-time image capture. For each test, 4 μL water droplets were gently deposited on the laser-processed areas to assess the wettability transition across different structural configurations.

## 3. Results and Discussion

### 3.1. Mineralogical Composition and Inhomogeneous Distribution in Green Schist

The green schist samples used in this study exhibit a fine- to medium-grained granoblastic and scaly metamorphic texture, with well-developed foliation. The mineralogical composition is heterogeneous and primarily includes quartz (~40%), chlorite (~35%), muscovite (~15%), biotite (~4%), carbonates (~4%), and minor dust-like carbonaceous matter (~2%). Quartz occurs as irregular granular crystals with particle sizes generally below 1 mm. It is colorless, exhibits extremely low relief, lacks cleavage or twinning, and is uniaxial positive. The distribution of quartz is uneven, indicating its origin from the recrystallization of detrital quartz grains during regional metamorphism. In contrast, chlorite appears as irregular flaky crystals, predominantly below 1 mm in size, though some grains exceed 1 mm. It is green, shows weak pleochroism, displays a first-order gray–white interference color, and exhibits nearly parallel extinction. Chlorite commonly forms lens-shaped or banded aggregates dispersed within quartz-rich regions, contributing significantly to the foliation of the rock.

Muscovite is typically flaky and fine-grained (<1 mm), though larger crystals exceeding 1 mm are occasionally observed. It is pale green, exhibits a second-order yellow–green interference color, parallel extinction, and positive elongation. Muscovite tends to occur within the chlorite-rich bands, often forming oriented aggregates aligned along the foliation plane. Similarly, biotite appears as flakes usually smaller than 1 mm, with some reaching up to 2.5 mm. It is brown with strong pleochroism and absorption, displays high second-order interference colors, parallel extinction, positive elongation, and a very small 2V angle. Biotite is also distributed within chlorite bands and contributes to the dark-toned streaks within the rock.

Furthermore, carbonates (including calcite and dolomite) are found as irregular granular crystals smaller than 1 mm. Some larger grains show rhombohedral cleavage and high relief, often displaying high-order white interference colors. These minerals tend to form irregularly distributed aggregates within chlorite-rich zones. Staining with alizarin red S indicates the presence of both calcite (stained red) and dolomite (unstained). Finally, carbonaceous matter exists as dust-like black particles irregularly distributed across the sample. It commonly aggregates into lens-shaped or banded dark regions mixed with chlorite, contributing to the overall heterogeneity of the green schist. Taken together, chlorite, muscovite, biotite, and carbonate minerals are interpreted as products of metamorphic co-crystallization from the original clay-rich sediments. The platy minerals, especially when aligned, form the well-developed foliated structure characteristic of the green schist and highlight its complex mineralogical heterogeneity.

In axially cut samples, however, the spatial distribution of minerals appears to be more random and less foliated compared to the transverse sections (Figure S1, in Supplementary S1, Supporting Information). Quantitative compositional analysis of such samples reveals that quartz dominates the matrix at approximately 75%. It exists as irregular grains mostly smaller than 1 mm, although some particles can reach up to 2 mm in size. These quartz crystals remain colorless, possess very low relief, exhibit no cleavage or twinning, and are uniaxial positive, indicating their origin as metamorphically recrystallized quartz sand grains. Chlorite in the axial section accounts for about 10%, occurring as irregular flakes with grain sizes mostly below 1 mm, although a few exceed this threshold. The chlorite is weakly pleochroic, with a first-order gray–white interference color and nearly parallel extinction, often forming discontinuous lens- or stripe-like clusters within the quartz matrix. Biotite (approximately 5%) is present as larger flakes, with individual grains reaching up to 2 mm. It is strongly pleochroic with distinct absorption, exhibits high second-order interference colors, and shows parallel extinction with positive elongation and a very small negative 2V angle. Biotite tends to be discontinuously distributed within quartz-dominated regions, contributing to localized dark streaks and further underscoring the compositional randomness of the axially oriented samples.

**Figure 2:**
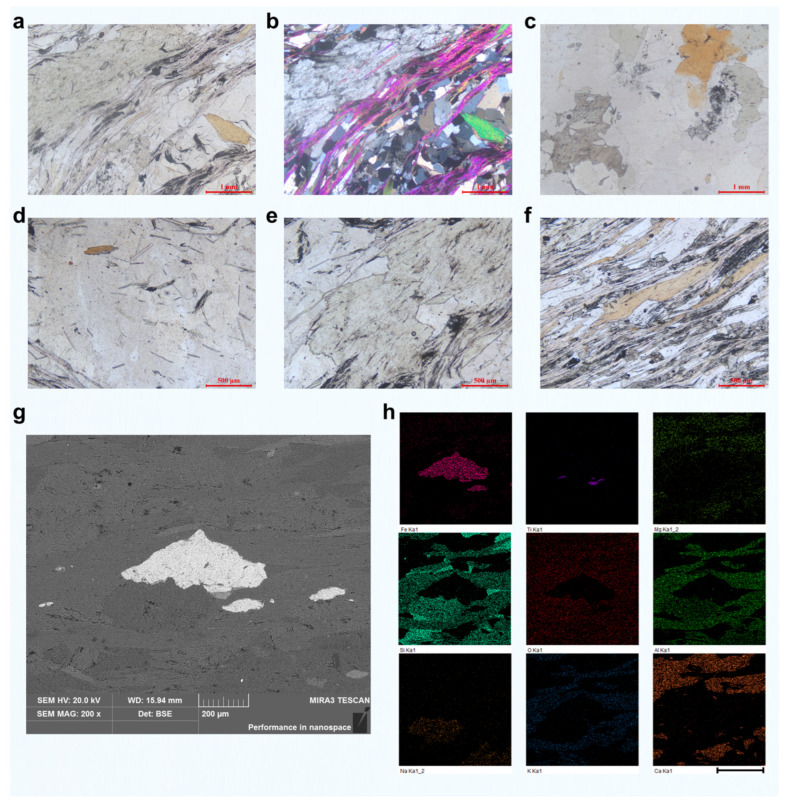
Composition analysis of green schist. Optical microscope analysis of the composition of transverse green schist under (**a**) plane polarized light and (**b**) crossed polarized light. (**c**) Optical microscope analysis of the composition of axial green schist under plane polarized light. Optical microscope image of (**d**) quartz, (**e**) chlorite, and (**f**) muscovite composition in transverse green schist. (**g**) BSE image of a selected surface area in transverse green schist, showing an embedded metal particle. (**h**) Elemental compositions and distributions in the corresponding area based on EDS analysis, mainly including Fe, Ti, Mg, Si, O, Al, Na, K, and Ca. Scale bar, 400 μm.

Given the clearer mineral alignment and more consistent structural orientation observed in the transversely cut green schist, all subsequent analyses and femtosecond laser grooving experiments were performed on these samples to ensure greater repeatability and comparability. To further illustrate the compositional heterogeneity of green schist, a representative microstructural region was selected for detailed analysis (as shown in Figure 2g). Elemental mapping of this area revealed the presence of localized Fe- and Ti-rich regions, surrounded by a matrix of Si, O, Mg, Al, K, and Ca. This elemental distribution clearly indicates a highly inhomogeneous composition, where metallic and silicate phases are intimately interwoven on the microscale.

From the perspective of the laser–matter interaction mechanism, this compositional variability plays a critical role in determining the outcome of femtosecond laser structuring. Metallic elements, such as Fe and Ti, tend to absorb femtosecond laser energy primarily through linear absorption processes. Their higher absorption coefficients lead to more intense energy deposition and, consequently, more pronounced ablation features [35]. In contrast, silicate minerals, like quartz (SiO_2_), primarily undergo nonlinear multiphoton absorption, especially given the ultrashort pulse duration and high peak intensities of femtosecond lasers. This nonlinear absorption is typically less efficient, resulting in lower ablation rates and more subtle surface modifications [36].

Such stark contrasts in absorption behavior among the various mineral constituents mean that femtosecond laser irradiation on green schist does not result in uniform material removal. Instead, it produces a highly heterogeneous surface morphology, with variable depths, microcrack formation, and irregular feature boundaries, all of which depend on the local phase composition and its optical and thermal response characteristics. This intrinsic complexity poses both challenges and opportunities for precision microstructuring. While it introduces difficulties in achieving uniform textures, it also opens the possibility of exploiting these differences to tailor localized surface features, potentially enabling functionally graded micro/nanostructures that can enhance surface properties, such as hydrophobicity.

### 3.2. Femtosecond Laser Processing Results on the Heterogeneous Composition of Green Schist

**Figure 3:**
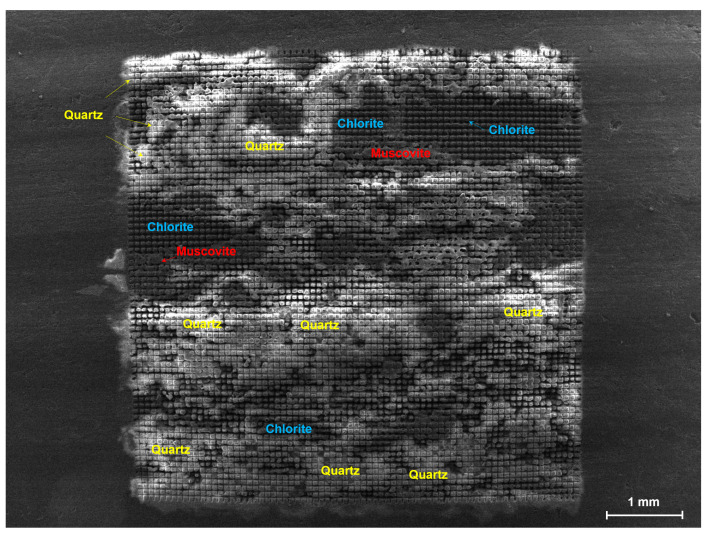
SEM image showing a typical result of femtosecond laser grooving on green schist. Based on the differences in electrical conductivity and laser absorption among the major components of green schist, the processed surface can be classified into three regions corresponding to quartz, chlorite, and muscovite, as indicated in the image.

In the practical femtosecond laser grooving experiments, a 5 mm × 5 mm area of green schist was selected as the processing region for fabricating microgrooves with varying spacing. Among the tested patterns, one representative result is shown in Figure 3, where microgrooves were produced simultaneously along both the horizontal and vertical directions with a spacing of 80 μm (two scan passes). This configuration provides a clear view of how the heterogeneous composition of green schist influences the morphology and uniformity of the laser-induced features across different crystallographic domains and mineral phases.

The green schist sample contains multiple mineral phases, including quartz, chlorite, and muscovite. The heterogeneous mineral composition leads to significant variations in the resulting laser-induced morphology. The groove morphologies varied markedly depending on the mineral type:

In quartz-rich regions, the grooves were relatively shallow with smoother but less sharply defined edges. This suggests limited absorption of femtosecond laser energy, likely due to the high hardness and low nonlinear absorption of quartz, which reduce the efficiency of material removal [37]. Chlorite regions, in comparison, exhibited considerably deeper and wider grooves. This behavior is attributed to the lower thermal conductivity and higher iron–magnesium content of chlorite, which enhance local heat accumulation and facilitate ablation. These regions show more pronounced microstructural damage, indicating that chlorite is the most laser-responsive phase. Muscovite-dominated areas were characterized by melting and resolidification. Groove edges appeared partially collapsed with some material redeposition, and smooth surface textures were observed in certain regions. These features suggest that muscovite tends to undergo thermally driven melting rather than explosive ablation, likely influenced by its layered structure and strong anisotropy [38].

According to the mineral distribution annotated in Figure 3, quartz accounts for approximately 50% of the selected area, primarily located in the lower middle and upper left parts. Chlorite makes up about 30%, mainly in the upper and lower middle regions, while muscovite comprises around 15%, scattered near the center. These compositional differences directly influence the spatial uniformity of the laser processing results. Furthermore, there is a clear relationship between mineral distribution and groove quality. In quartz-dominant zones, the grooves are consistently aligned and uniform in depth. In contrast, areas with mixed or alternating chlorite and muscovite show more irregular features, including overlapping grooves, misalignment, and signs of local defocusing. These findings highlight the strong influence of microscale compositional heterogeneity on laser–material interaction behavior.

**Figure 4:**
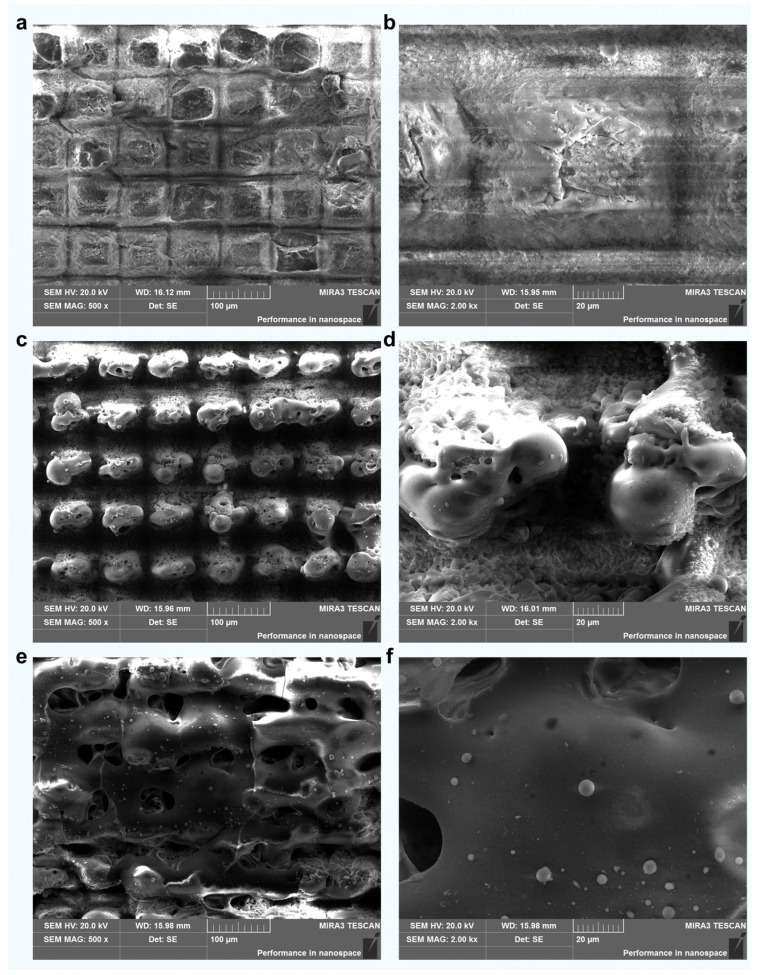
Typical morphology analysis and comparison of the three major components (quartz, chlorite, and muscovite) under femtosecond laser grooving. (**a**,**b**) Quartz surface after laser processing. (**c**,**d**) Chlorite surface after laser processing. (**e**,**f**) Muscovite surface after laser processing.

To investigate the femtosecond laser response of different mineral components within green schist, representative morphologies of quartz, chlorite, and muscovite after laser grooving are presented in Figure 4. The scanning sequence consisted of a longitudinal pass followed by a transverse pass. The preliminary longitudinal scan introduced shallow pits, which altered the energy deposition and material removal dynamics during the subsequent transverse scan. This resulted in increased ablation volumes, especially in the quartz and chlorite regions, indicating a cumulative effect induced by the scanning strategy.

**Figure 5:**
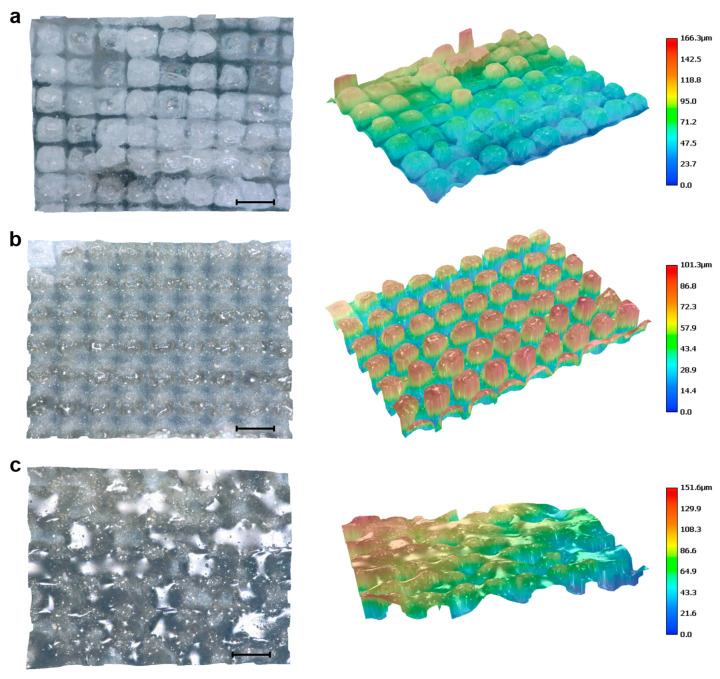
Three-dimensional surface morphology of different components of green schist after femtosecond laser grooving. (**a**) Quartz surface after laser processing. (**b**) Chlorite surface after laser processing. (**c**) Muscovite surface after laser processing. Scale bar, 100 μm.

Quartz-rich regions displayed relatively regular ablation morphologies. In areas between adjacent grooves, large-scale flaking occurred in the non-irradiated zones, suggesting the presence of brittle fracture induced by thermal or mechanical stress (Figure 4a,b). The directly irradiated surfaces featured rectangular-shaped residual structures with localized microcracks, indicating a non-thermal, stress-dominated removal mechanism [39]. Due to its transparency and low linear absorption at the laser wavelength, quartz primarily underwent multiphoton absorption, leading to moderate material removal. The resulting groove widths were approximately 30–40 μm transversely and 50–60 μm longitudinally, with overall depths around 40–50 μm (Figure 5a), characteristic of cold ablation with relatively low efficiency.

In contrast, chlorite exhibited a markedly higher ablation efficiency. Its surface morphology included fine ablation textures at the bottom of the laser-processed zones and signs of melting at the top, implying a combination of cold and thermally assisted ablation (Figure 4c,d). The longitudinal scan produced discontinuous grooves spaced by 10–20 μm, while the transverse scan formed more continuous grooves measuring 60–80 μm in width. The maximum etching depth reached 90–100 μm (Figure 5b). The enhanced ablation performance is attributed to the presence of Fe and Mg in chlorite, which increase linear absorption and facilitate energy accumulation during laser exposure. Additionally, the mineral's layered structure and relatively low thermal conductivity may have promoted localized heating, further enhancing material removal.

Muscovite, on the other hand, responded to femtosecond laser irradiation primarily through thermal effects. Significant melting, flow, and microscale sputtering features were observed. Spherical re-solidified particles with diameters of 2–4 μm were present on the melted surface, likely resulting from laser-induced splashing followed by rapid cooling and redeposition [40]. The ablated surface displayed random micro-pits and a generally smooth, flattened morphology dominated by post-melting flow (Figure 4e,f). The etching depth varied across the region, with no obvious periodicity or groove regularity, reflecting the material’s structural anisotropy and its tendency toward layer delamination under thermal stress (Figure 5c).

Overall, the morphological differences observed across the three mineral components reflect the interplay between laser parameters, material properties, and ablation mechanisms. Cold ablation dominated in quartz, thermal-assisted ablation prevailed in chlorite, and melting-redeposition effects were prominent in muscovite. These findings highlight the selective response of green schist’s heterogeneous microstructure under femtosecond laser irradiation and provide insights into the controllability of laser-induced modifications at the microscale.

The preceding analysis was primarily based on SEM imaging, combined with the response characteristics of different mineral phases under femtosecond laser irradiation. Among these phases, quartz could be visually identified due to its low electrical conductivity, which typically results in a bright contrast in SEM images. However, for other mineral components, no clear image features or supporting compositional data were available. To more directly reveal how different phases respond to femtosecond laser grooving, particularly to identify the specific mineral type responsible for the observed grooving morphologies and to clarify the elemental distribution on the surface after laser interaction, we conducted systematic EDS analysis on representative regions.

**Figure 6:**
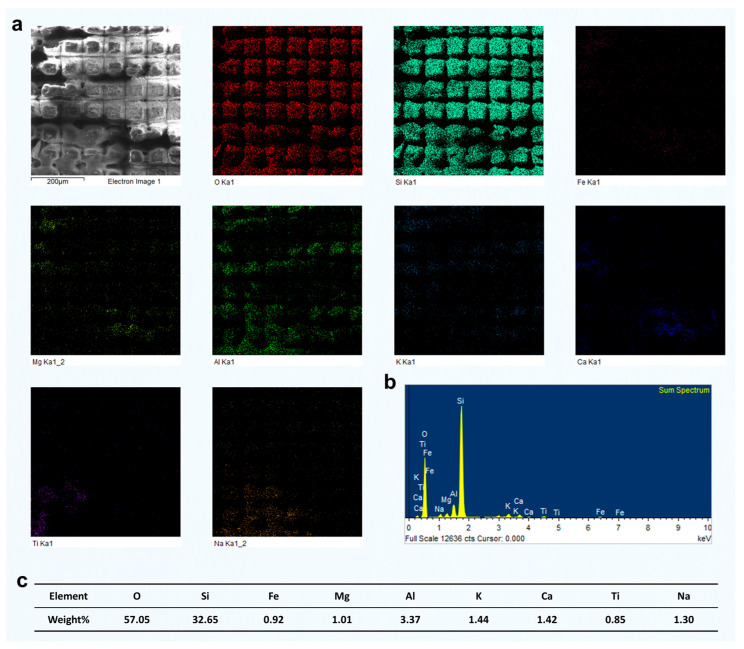
Elemental mapping and EDS analysis of the green schist with quartz as the major component after femtosecond laser grooving. (**a**) SEM image of the ablated area and corresponding elemental distribution maps (O, Si, Fe, Mg, Al, K, Ca, Ti, and Na). (**b**) EDS spectrum shows a strong Si peak, confirming SiO_2_-rich matrix with traces of muscovite components. (**c**) Quantitative elemental composition in weight percent, with O and Si being the most abundant, consistent with typical quartz composition.

We selected a region primarily composed of quartz with a minor muscovite content and analyzed the elemental distribution after laser grooving using EDS (Figure 6). The results showed an O content of 57.05 wt%, Si of 32.65 wt%, and Al of 3.37 wt%. The combined content of K, Ca, and Na was ~1.3–1.4 wt%, while Fe and Mg were present at ~0.9 wt% and 1.0 wt%, respectively, indicating trace levels. The dominant presence of Si and O, with a weight ratio close to that of pure quartz (SiO_2_), suggests that this region is primarily composed of quartz. The presence of minor Al and K indicates that small amounts of muscovite or other mica-group minerals may exist as secondary phases. Trace levels of Fe and Mg could originate from minor chlorite or altered biotite, but their overall contribution is negligible. These EDS results confirm that the analyzed area represents a quartz-rich microdomain, likely corresponding to a quartz vein or quartz layer within the green schist structure.

Furthermore, it is important to note that after femtosecond laser grooving, the distribution of O and Si elements in the remaining material was still relatively uniform. This indicates that the femtosecond laser grooving process did not significantly alter the elemental composition of the original mineral phases. The minimal disturbance in elemental distribution suggests that femtosecond laser processing mainly alters the surface morphology, with little impact on the bulk chemical composition. This supports the conclusion that the laser's effects are confined to the surface layer. This observation is crucial as it demonstrates that femtosecond laser grooving can be a controlled process that preserves the inherent material properties of the untouched regions.

**Figure 7:**
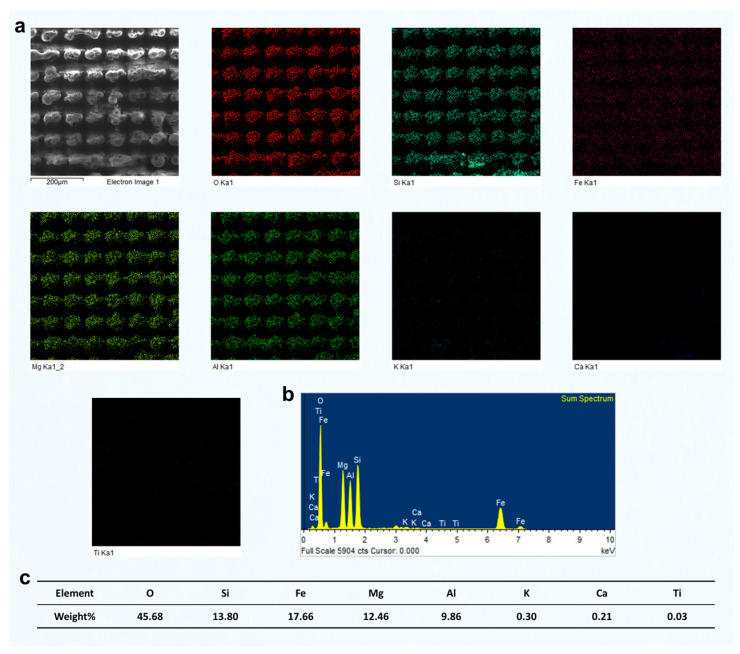
Elemental mapping and EDS analysis of the green schist with chlorite as the major component after femtosecond laser grooving. (**a**) SEM image of the ablated area and corresponding elemental distribution maps (O, Si, Fe, Mg, Al, K, Ca, and Ti). (**b**) EDS spectrum showing O, Fe, Mg, Si, and Al as dominant elements. (**c**) Quantitative elemental composition in weight percent, with O being the most abundant, followed by Si, Fe, and Mg, consistent with typical chlorite composition.

After femtosecond laser grooving, the chlorite-rich region exhibited significant responses to laser irradiation. EDS analysis revealed relatively high contents of Fe, Mg, and Al, confirming that this area was primarily composed of chlorite (Figure 7). As a Fe- and Mg-rich phyllosilicate, chlorite strongly absorbs laser energy, which resulted in the formation of distinctly deeper grooves after processing. In comparison, the quartz-rich region showed much weaker responses. That area had a high Si and O content, and its surface morphology remained largely unchanged after laser treatment. This difference can be attributed to the absence of Fe and Mg, which are key contributors to enhanced energy absorption and material removal under femtosecond laser irradiation. The relatively high Al content further supports the identification of chlorite as the dominant mineral in the analyzed area. Since Al is commonly present in the structural units of chlorite, the observed grooving features are consistent with its known response behavior. In contrast to quartz, chlorite showed much more evident laser-induced modifications, including deeper ablation features and surface restructuring.

**Figure 8:**
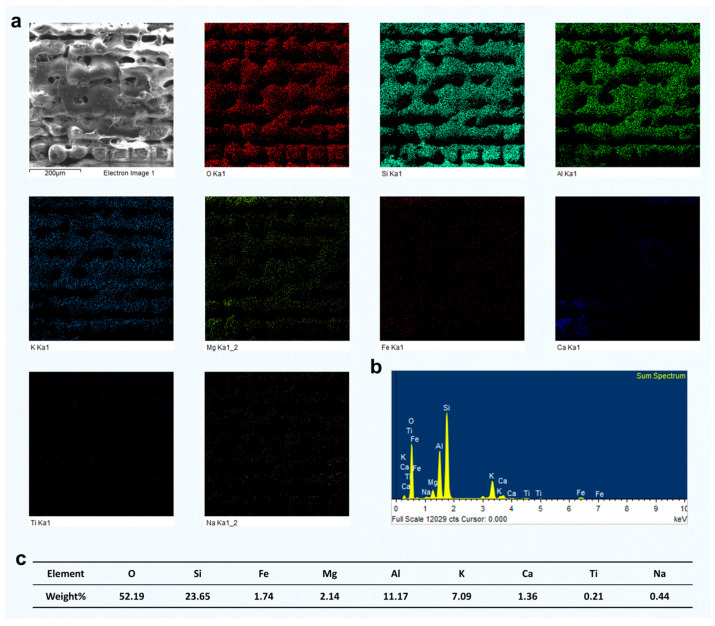
Elemental mapping and EDS analysis of the green schist with muscovite as the major component after femtosecond laser grooving. (**a**) SEM image of the ablated area and corresponding elemental distribution maps (O, Si, Fe, Mg, Al, K, Ca, Ti, and Na). (**b**) EDS spectrum of the scanned region. (**c**) Elemental composition (weight %) of the selected area.

The EDS analysis of the region identified as muscovite-rich is shown in Figure 8. The elevated levels of K (7.09 wt%) and Al (11.17 wt%), together with a significant amount of Si (23.65 wt%), strongly indicate the presence of muscovite, a typical potassium aluminum silicate mineral. Muscovite has a representative formula of Kal_2_(AlSi_3_O_10_)(OH)_2_, and is commonly found in metamorphic rocks, such as green schist. The high Si content also suggests the presence of quartz, which is frequently intergrown with muscovite in these rocks. The relatively low concentrations of Mg (2.14 wt%) and Fe (1.74 wt%) are within the typical range for natural muscovite, where minor isomorphic substitution of Fe and Mg can occur. The presence of Ca (1.36 wt%) and Na (0.44 wt%) is minor, likely reflecting trace feldspar or carbonate phases. Ti (0.21 wt%) is present only in trace amounts, indicating the absence of significant Ti-bearing minerals.

Overall, the composition indicates that this region is mainly a muscovite–quartz domain, typically found in micaceous layers or foliation zones of green schist. Compared with the previously analyzed chlorite-rich and quartz-rich areas, this region stands out for its high K and Al content and likely exhibits a more distinct platy or flaky microstructure.

### 3.3. Effect of Groove Spacing on Groove Depth and Surface Morphology

**Figure 9:**
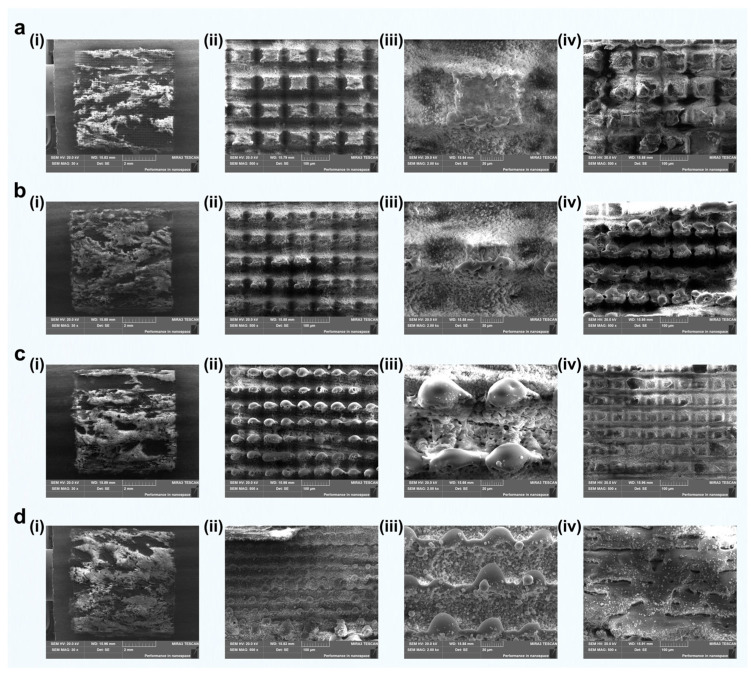
Comparison of green schist surface structures processed by femtosecond laser grooving with different groove spacings. The structure was processed using a single scan. (**a**) A groove spacing of 100 μm. (**b**) A groove spacing of 80 μm. (**c**) A groove spacing of 60 μm. (**d**) A groove spacing of 40 μm. (**i**) shows the overall morphology of the femtosecond laser-grooved region, (**ii**,**iii**) show the surface morphology of the chlorite-rich region after femtosecond laser grooving, (**iv**) shows the surface morphology of the quartz or muscovite-rich region after femtosecond laser grooving.

During femtosecond laser grooving on green schist, using a laser spot diameter of ~30 μm, changes in groove pitch (100 μm, 80 μm, 60 μm, and 40 μm) had a notable effect on laser–material interactions (Figure 9). This effect was particularly evident in regions dominated by quartz, chlorite, and muscovite. At large pitches, such as 100 μm (Figure 9a(i)), the laser tracks remained separate, resulting in minimal overlap and primarily isolated ablation events. Under these conditions, the inherent differences in laser absorption among mineral phases became more distinct. For instance, chlorite was more susceptible to localized melting or ablation (Figure 9a(ii),(iii)), while quartz typically showed minimal modification and retained a smooth surface (Figure 9a(iv)). 

As the pitch was reduced to 80 μm and especially to 40 μm (Figure 9b–d), it caused the laser–material interaction to shift from isolated ablation to cumulative effects, including thermal buildup and intensified nonlinear interactions [41]. Such overlapping often induced phase transitions, microstructural rearrangements, or resolidification and redeposition of ablated material. From the surface morphology of chlorite under femtosecond laser grooving with different pitches, when the pitch was 100 μm, the laser ablation behavior was primarily characterized by typical cold ablation (Figure 9a(ii)), resulting in well-defined and relatively deep grooves [42]. This suggests that laser energy was concentrated on each individual track with minimal impact on surrounding areas. However, high-magnification SEM images revealed partial melting in localized regions, indicating thermal accumulation in some areas even under single-track ablation (Figure 9a(iii)). 

As the pitch decreased to 80 μm, the laser tracks remained independent, but the unablated areas between the tracks were significantly reduced, leading to increased overall surface coverage (Figure 9b(ii)). At this pitch, the unablated areas of chlorite further decreased, and more prominent melting boundaries were observed around some tracks, indicating that the thermal influence began to extend to adjacent tracks (Figure 9b(iii),(iv)). When the pitch was further reduced to 60 μm, the laser tracks still did not overlap, but with an increased coverage density, chlorite exhibited noticeable thermal melting in multiple regions. This enhanced thermal effect suggests that the energy deposition from the laser was sufficient to cause significant modification of the material, even without track overlap (Figure 9c(ii),(iii)). At a pitch of 40 μm, the laser almost covered the entire surface of chlorite (Figure 9d(ii)). Although the tracks did not completely overlap, the thermal influence zones were highly coupled, resulting in continuous melting and vaporization behaviors. At this point, the original structure of chlorite was almost completely destroyed, leaving only melted particles approximately 10 μm in diameter on the surface, indicating significant thermal accumulation (Figure 9c(iii)).

In contrast, other mineral components, such as quartz and muscovite, exhibited greater structural stability under the same pitch variations. Quartz, with its lower laser absorption rate, showed stable removal with a relatively low ablation efficiency at all pitch settings, with smooth melting or slight ablation on the surface (Figure 9a(iv),c(iv)). Muscovite, with medium laser absorption and a large thermal expansion coefficient, exhibited melting at higher coverage conditions, filling the grooves and forming a relatively smooth surface (Figure 9d(iv)). Therefore, chlorite, quartz, and muscovite showed significant differences in their responses to femtosecond laser ablation at different pitches. Quartz, with its low absorption and high thermal stability, was relatively insensitive to pitch variation and usually maintained a smooth, slightly melted appearance (Figure 9c(iv)). Chlorite, by contrast, had higher absorption and moderate thermal stability, making it highly sensitive to smaller pitches (Figure 9c(ii),(iii)). This sensitivity often resulted in overheating, local collapse of the melted regions, and damage along the groove edges. Muscovite displayed moderate absorption and thermal stability but also exhibited relatively high thermal expansion and a distinct layered structure. In areas with high scan overlap, muscovite not only underwent localized ablation but also tended to melt and flow, producing filled grooves along with delamination or debris formation. These results provide important insights into the laser interaction mechanisms and parameter optimization for heterogeneous mineral systems.

In summary, decreasing the groove pitch increased the differential response of mineral phases to femtosecond laser irradiation. Surface morphology evolved from isolated grooves to continuous modification patterns, shaped by the specific photothermal properties of each mineral. With constant laser parameters, the choice of groove pitch directly influenced material removal, energy deposition uniformity, and the thermal–mechanical response of different regions.

**Figure 10:**
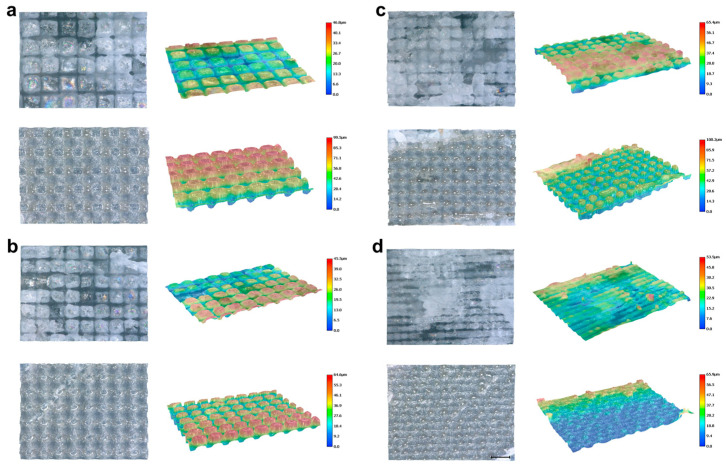
Three-dimensional surface morphology of different components of green schist after femtosecond laser grooving with different groove spacings. The figure mainly illustrates the femtosecond laser-induced surface features of quartz (upper figure) and chlorite-rich (lower figure) region. The structure was processed using a single scan. (**a**) A groove spacing of 100 μm. (**b**) A groove spacing of 80 μm. (**c**) A groove spacing of 60 μm. (**d**) A groove spacing of 40 μm. Scale bar, 100 μm.

We further measured the three-dimensional surface morphology of quartz and chlorite under different groove pitches, as shown in Figure 10. At a pitch of 100 μm, both the quartz and chlorite regions exhibited relatively regular rectangular structures. The maximum ablation depth in the quartz region reached 46.8 μm, while in the chlorite region it reached 99.5 μm (Figure 10a). This result indicated that, under femtosecond laser irradiation, the removal depth of quartz due to nonlinear absorption was less than half the removal depth of chlorite, which was governed by linear absorption. Additionally, the groove width in the quartz region was approximately 10–20 μm, whereas that in the chlorite region approached 50 μm. Notably, as shown in Figure 10a, grooves formed by longitudinal scanning were much shallower than those formed by transverse scanning. At the intersections of the two scanning directions, where the material was ablated twice, the groove depth reached its maximum. This phenomenon was observed in both the quartz and chlorite regions.

As the scanning pitch decreased from 100 μm to 80 μm, 60 μm, and 40 μm (Figure 10b–d), the groove depth in the quartz region remained nearly unchanged. More precisely, the depth in quartz did not show a significant trend of increase or decrease with decreasing pitch, indicating a stable response to changes in scanning density. In contrast, the chlorite region showed increases in both groove width and depth due to enhanced thermal effects, which was consistent with the surface morphology shown in Figure 9. However, the actual depth measured on the processed surface decreased instead. This indicated that part of the initial chlorite surface was vaporized or peeled off during femtosecond laser ablation, resulting in a reduced final structure height. This effect became more pronounced at a 40 μm pitch, where uniformly distributed molten dot-like structures with a height of approximately 10 μm were formed on the surface (Figure 10d).

These results highlighted the distinct ablation responses of quartz and chlorite under varying scanning pitches. While quartz exhibited consistent and limited morphological changes due to its lower laser absorption, chlorite responded sensitively to pitch reduction, with increasing thermal effects leading to structural melting, material removal, and the formation of residual molten features. This contrast offered important insight for optimizing femtosecond laser grooving parameters and regulating the surface hydrophobic behavior of the material in subsequent applications.

Based on the understanding of the effect of different groove pitches on the components of green schist, we further analyzed the surface elemental distribution characteristics of different green schist components after femtosecond laser ablation at various groove pitches. In the previous study, we performed a detailed investigation on the green schist sample with a groove pitch of 80 μm and two scanning passes. The elemental content and distribution patterns of quartz, chlorite, and muscovite after femtosecond laser ablation were clearly identified. Building on this, we selected the two-pass scanning condition and compared the elemental changes in the surface composition of different mineral components formed under different groove pitches.

**Figure 11:**
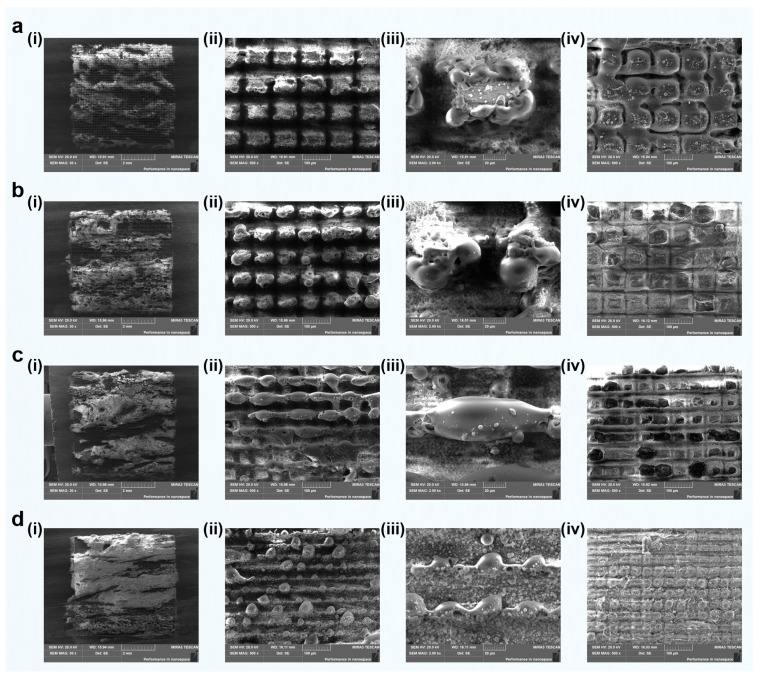
Comparison of green schist surface structures processed by femtosecond laser grooving with different groove spacings. The structure was processed using two scans. (**a**) A groove spacing of 100 μm. (**b**) A groove spacing of 80 μm. (**c**) A groove spacing of 60 μm. (**d**) A groove spacing of 40 μm. (**i**) shows the overall morphology of the femtosecond laser-grooved region, (**ii**,**iii**) show the surface morphology of the chlorite-rich region after femtosecond laser grooving, (**iv**) shows the surface morphology of the quartz or muscovite-rich region after femtosecond laser grooving.

Figure 11 shows the groove formation results on green schist under different groove pitches with two passes of femtosecond laser scanning. Our primary focus is on the morphological changes in the chlorite and quartz components of green schist after femtosecond laser grooving. Compared to Figure 9, under the two-pass scanning conditions, the micro-groove depth in the chlorite component significantly increased (in Figure 11a(ii),b(ii)), the grooves formed in the chlorite component after transverse scanning with femtosecond laser no longer show clear bottoms, whereas the etching texture in these regions is more distinct in Figure 9). Additionally, the thermal effect generated by femtosecond laser under two-pass scanning was also enhanced. From the two-pass results at an 80 μm pitch (Figure 11c(ii)), the melting behavior observed was already similar to that seen with a single pass at a 60 μm pitch.

In studies of laser–material interaction mechanisms, it is theorized that after the laser induces various microstructures on the material surface, it increases the material's absorption capacity for subsequent laser pulses [43]. Therefore, under the two-pass scanning conditions, the groove depth further increased, and the enhanced laser absorption intensified the thermal effects generated by the femtosecond laser. At a 60 μm scanning pitch, we observed that melted areas on the chlorite surface were significantly larger than those from a single pass. This indicates that increased laser absorption heated and melted more chlorite components, leading to deeper and wider thermal effects (Figure 11c(ii),(iii)). 

Under femtosecond laser ablation, two-pass scanning revealed that decreasing the scanning pitch significantly increased the area of quartz surface peeled off by laser-induced stress or shock. This indicates that closer scan lines intensify mechanical effects on quartz, leading to more extensive surface peeling. At scanning pitches of 80 μm and 60 μm, more peeling was observed on the quartz surface (Figure 11b(iv),c(iv)). At a scanning pitch of 40 μm, melting was also observed on the quartz surface (Figure 11d(iv)).

**Figure 12:**
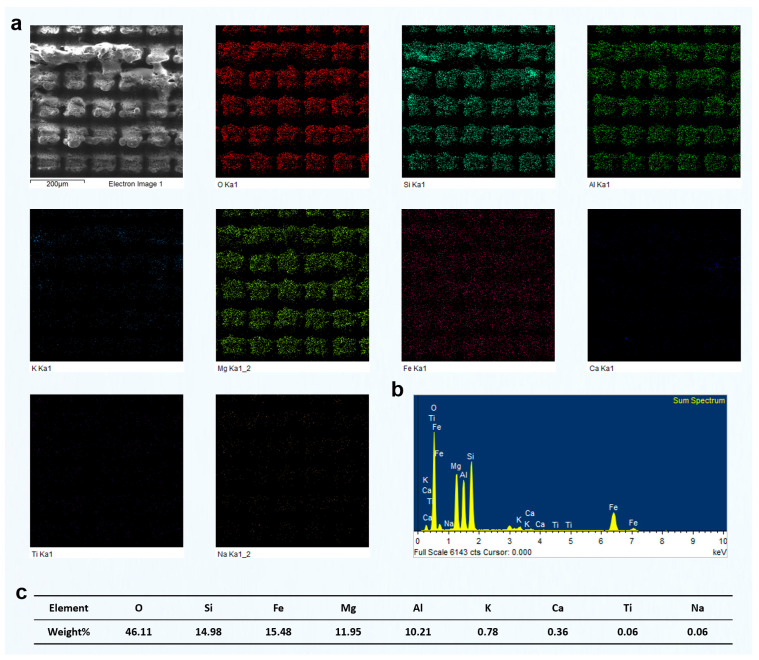
Elemental mapping and EDS analysis of the green schist with chlorite as the major component after femtosecond laser grooving. A groove spacing of 100 μm was used and the structure was processed using two scans. (**a**) SEM image of the ablated area and corresponding elemental distribution maps (O, Si, Fe, Mg, Al, K, Ca, Ti, and Na). (**b**) EDS spectrum showing O, Fe, Mg, Si, and Al as dominant elements. (**c**) Quantitative elemental composition in weight percent, with O being the most abundant, followed by Si, Fe, and Mg, consistent with typical chlorite composition.

Figure 12 shows the results of two-pass femtosecond laser grooving at a scanning pitch of 100 μm. The surface in this region is mainly composed of O, Si, Fe, Mg, and Al, corresponding to chlorite. According to the EDS mapping results, the surface elemental distribution remained relatively uniform after laser ablation, with no evident redistribution of elements induced by the process. Furthermore, typical melted regions observed in the SEM images did not exhibit significant changes in elemental composition. Compared with the results at an 80 μm pitch shown in Figure 7, the concentrations of O, Si, Fe, Mg, and Al remained largely unchanged. Although the increased pitch enlarged the area not directly processed by the laser, the elemental composition and distribution within the same observation range showed minimal variation. This indicates that femtosecond laser processing had limited influence on unprocessed areas, preserving the original elemental proportions. In addition, unlike the 80 μm pitch results, the EDS mapping at 100 μm showed a larger area of unprocessed chlorite, which also included some partially melted regions and exhibited a distinct square-shaped pattern.

**Figure 13:**
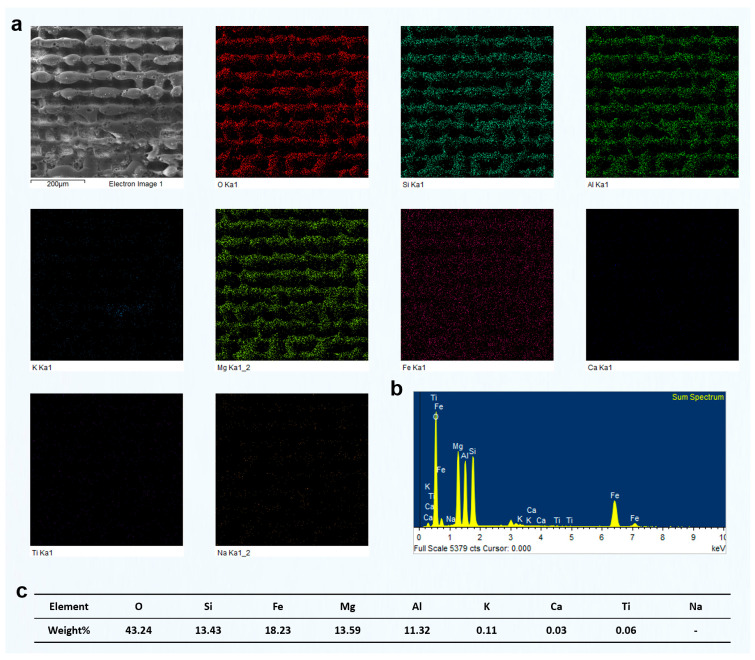
Elemental mapping and EDS analysis of the green schist with chlorite as the major component after femtosecond laser grooving. A groove spacing of 60 μm was used and the structure was processed using two scans. (**a**) SEM image of the ablated area and corresponding elemental distribution maps (O, Si, Fe, Mg, Al, K, Ca, Ti, and Na). (**b**) EDS spectrum showing O, Fe, Mg, Si, and Al as dominant elements. (**c**) Quantitative elemental composition in weight percent, with O being the most abundant, followed by Si, Fe, and Mg, consistent with typical chlorite composition.

**Figure 14:**
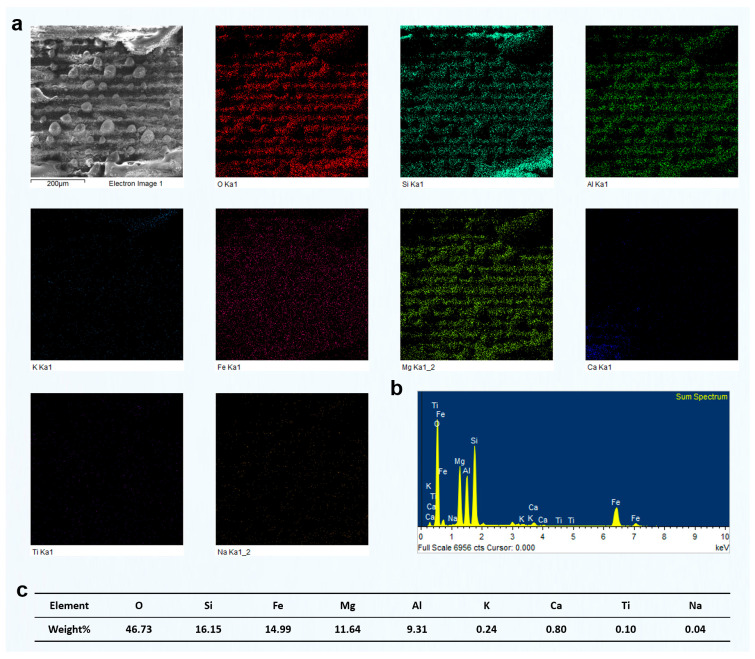
Elemental mapping and EDS analysis of the green schist with chlorite as the major component after femtosecond laser grooving. A groove spacing of 40 μm was used and the structure was processed using two scans. (**a**) SEM image of the ablated area and corresponding elemental distribution maps (O, Si, Fe, Mg, Al, K, Ca, Ti, and Na). (**b**) EDS spectrum showing O, Fe, Mg, Si, and Al as dominant elements. (**c**) Quantitative elemental composition in weight percent, with O being the most abundant, followed by Si, Fe, and Mg, consistent with typical chlorite composition.

Similarly, we conducted EDS measurements on the surface of chlorite processed using two-pass femtosecond laser scanning at groove pitches of 60 μm and 40 μm. The results are presented in Figure 13 and Figure 14. As the scanning pitch decreased, the corresponding increase in scanning density led to a significantly higher number of laser pulses per unit area. This, in turn, intensified the cumulative thermal effects, raised the local surface temperature, and enhanced the likelihood of chlorite melting or vaporization. As a result, more prominent surface melting features were observed. At a pitch of 40 μm, repeated laser irradiation in the same area generated significant thermal and mechanical stress. This stress caused partial material removal through vaporization or laser-driven ejection. This led to a noticeable reduction in the final groove depth, which is contrary to the typical expectation of deeper ablation with increased energy input.

However, EDS surface scans indicated that the elemental composition of the chlorite region remained relatively consistent across different groove pitches, even after exposure to femtosecond laser processing. This suggests that, although the shorter pitch significantly intensified the thermal effects and caused melting or material removal on the chlorite surface, the overall elemental distribution remained stable without substantial compositional variation.

Moreover, in the results of femtosecond laser processing of chlorite at a 40 μm pitch (shown in the upper and lower positions of the SEM image in Figure 14), we observed higher contents of O and Si elements in this region, while other elements were present at lower concentrations. Based on the elemental distribution and SEM images, this region is identified as quartz. The quartz in this area has clearly undergone melting under femtosecond laser irradiation, with the extent of melting being significantly greater than that observed in the chlorite region. This highlights the importance of optimizing the groove pitch in femtosecond laser microprocessing. The groove pitch not only determines the state of different components under laser irradiation, but also influences the final surface morphology. The distribution of different components in green schist further dictates the specific morphological features achievable after femtosecond laser processing.

These observations highlight the spatial precision and selectivity of femtosecond laser microprocessing. Even under elevated energy conditions, energy deposition remains highly localized, which helps prevent widespread thermal damage in adjacent regions. Moreover, the observed reduction in groove depth implies that non-thermal mechanisms, such as plasma expansion caused by laser excitation, mechanical stress buildup, and subsequent layer separation, may become dominant when the scanning density is high. These processes are in line with the ultrafast and nonequilibrium nature of femtosecond laser interactions with matter. 

### 3.4. Effect of Scan Pass Number on Groove Depth and Surface Morphology

Quartz is the predominant component in green schist, and its surface morphological evolution under femtosecond laser grooving exhibits distinct trends depending on the groove pitch. At a pitch of 100 μm, due to the relatively small laser spot size (~30 μm) and the insufficient thermal accumulation associated with low-repetition femtosecond laser pulses, the groove width remains limited. As a result, a large portion of the quartz surface retains its original morphology after laser scanning. In contrast, at a reduced pitch of 40 μm, repeated laser irradiation leads to evident melting on the quartz surface, forming elongated molten features that result in an unstable microstructure.

To gain further insights into the effect of scanning parameters on surface morphology, we analyzed the microstructures formed at groove pitches of 80 μm and 60 μm under varying numbers of scan passes (Figures S2–S5). At an 80 μm pitch with six scan passes, portions of the quartz surface remained unablated, indicating that the laser coverage was still incomplete. However, at a pitch of 60 μm, the laser spot size and scanning interval were well matched, enabling more uniform energy deposition. Under these conditions, quartz exhibited a well-defined conical structure after grooving, which is highly suitable for fabricating hydrophobic micro- and nanostructures.

**Figure 15:**
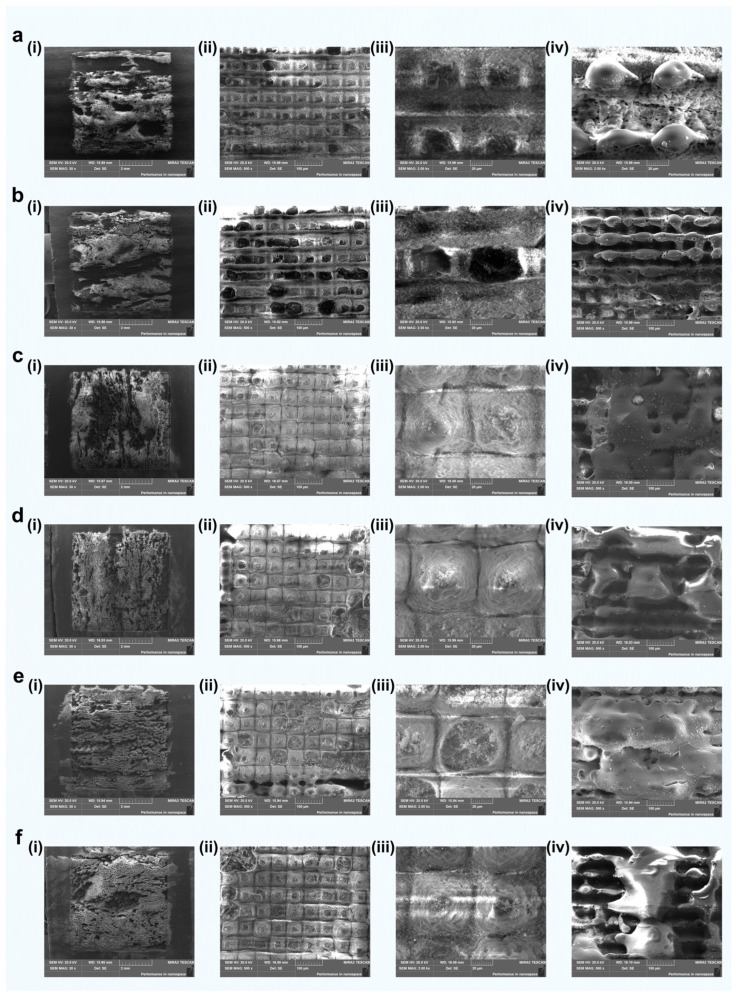
Results of femtosecond laser processing on green schist with a groove spacing of 60 µm under different scanning passes. (**a**) One scanning pass. (**b**) Two scanning passes. (**c**) Four scanning passes. (**d**) Six scanning passes. (**e**) Eight scanning passes. (**f**) Ten scanning passes. (**i**) shows the overall morphology of the femtosecond laser-grooved region, (**ii**,**iii**) show the surface morphology of the quartz-rich region after femtosecond laser grooving, (**iv**) shows the surface morphology of the chlorite or muscovite-rich region after femtosecond laser grooving.

Therefore, in subsequent analyses of the influence of scan pass number on grooving performance, we will focus primarily on the 60 μm groove pitch. This will allow a more detailed investigation into how femtosecond laser parameters govern the formation of micro- and nanostructures on green schist surfaces. The surface morphologies obtained under varying scan pass numbers (1, 2, 4, 6, 8, and 10 passes) are presented in Figure 15. In this analysis, we mainly focus on the morphological changes of the quartz component under laser irradiation, while also considering the responses of other key minerals, like chlorite and muscovite. This integrated approach allows for a more comprehensive understanding of the behavior of multiphase mineral systems under femtosecond laser processing.

The morphological evolution of quartz during femtosecond laser grooving at different scanning passes was generally categorized into three distinct patterns. At low scanning passes (1–2 passes), the groove morphology largely retained the original surface texture of the quartz. The grooves appeared as shallow, rectangular depressions, suggesting that the laser energy was insufficient to induce substantial surface restructuring. This was attributed to the relatively large scan pitch of 60 μm, which was twice the laser spot diameter (~30 μm), resulting in minimal overlap between adjacent scans. Under such limited exposure, thermal accumulation was minimal, and ablation was confined to a narrow area. Only a small amount of material was removed, without causing melting or morphological changes. These features were evident in the SEM images (Figure 15a,b(ii,iii)), where the quartz surface exhibited preserved bulk characteristics with only faint signs of ablation.

As the number of scanning passes increased to 4–6, the quartz surface underwent repeated irradiation, which enhanced both energy deposition and localized thermal effects. This promoted the formation of well-defined conical structures. Upon closer inspection, these cones displayed fine microtextures ranging from 1 to 2 μm on their surfaces. Together, the microscale cones and nanoscale features constituted a hierarchical micro/nanostructure. Prior studies have demonstrated that such composite textures can entrap air pockets, thereby enhancing surface hydrophobicity. The SEM images (Figure 15c,d(ii,iii)) confirmed this trend by showing conical structures with distinct boundaries and regular surface patterns. These features indicate efficient energy accumulation and stable morphological development under moderate scanning conditions.

When the scanning passes were further increased to 8–10, the previously formed conical structures became significantly enlarged and deepened. However, their structural integrity began to deteriorate. Signs of block-like delamination were already observable at the sixth pass, and this damage became increasingly pronounced with higher passes. As quartz is a brittle material, repeated high-energy laser exposure introduced excessive thermal and mechanical stresses, especially in deeper groove regions. The interaction between femtosecond laser pulses and the material likely triggered strong plasma shockwaves and thermal stress, both of which contributed to crack initiation and propagation. Consequently, the conical features began to fracture and peel away from the surface, compromising the stability and functionality of the micro/nanostructures. This degradation process was clearly captured in Figure 15e,f(ii,iii), where the cone structures appeared fractured, irregular, and partially detached.

In addition to the quartz-rich regions, the chlorite- or muscovite-rich areas, shown in Figure 15(iv), also underwent notable morphological evolution. At low scanning passes, these surfaces showed only minor texturing with limited melting or ablation. With increased scanning (4–6 passes), shallow pits and signs of localized melting began to emerge, indicating moderate heat accumulation and phase transitions. However, beyond six passes, the chlorite/muscovite regions exhibited extensive thermal damage, including molten-like globular morphologies and porous textures (Figure 15f(iv)). These changes suggested that although these phases initially tolerated laser exposure, they became more vulnerable to thermal softening and gas release at higher energy densities. Such degradation may adversely affect surface uniformity and reduce the stability of the modified surface for functional applications.

Overall, these results highlighted the critical role of scanning pass optimization in femtosecond laser processing. A moderate number of passes (approximately 4–6) effectively facilitated the formation of stable, functional conical microstructures in quartz while preserving the integrity of surrounding mineral components. In contrast, excessive scanning led to surface degradation, structural delamination, and thermal damage, ultimately compromising the long-term performance and robustness of the textured surfaces.

**Figure 16:**
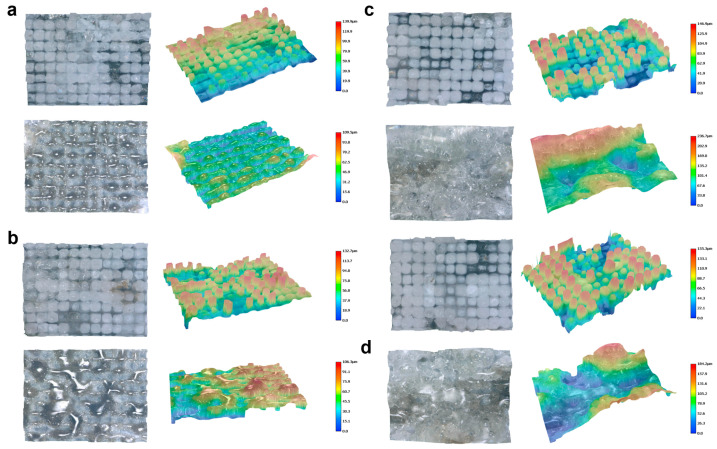
Three-dimensional surface morphology of different components of green schist after femtosecond laser grooving with different scanning passes. A groove spacing of 60 μm. (**a**) A scanning pass number of 2. (**b**) A scanning pass number of 4. (**c**) A scanning pass number of 6. (**d**) A scanning pass number of 8. Scale bar, 100 μm.

To further elucidate the morphological evolution induced by femtosecond laser grooving, three-dimensional surface morphologies of green schist were examined at different scanning passes, as shown in Figure 16. Each subfigure depicts both quartz-rich (top row) and chlorite-rich (bottom row) regions under the same processing parameters except for the scanning pass number (2, 4, 6, and 8 in Figure 16a–d, respectively).

At a low scanning pass number of 2 (Figure 16a), both quartz and chlorite surfaces exhibited shallow grooves with minimal structural transformation. The quartz region retained its original topography, forming faint rectangular depressions with an average depth below 130 μm. Similarly, the chlorite region displayed a slightly roughened texture, with depths not exceeding 100 μm. These results confirmed that limited pass numbers and large groove spacing (60 μm) resulted in weak energy overlap and negligible thermal accumulation in the quartz. Under such conditions, material removal was dominated by direct ablation with little melting or restructuring. When the scanning passes increased to 4 (Figure 16b), a distinct divergence in response between the two components began to emerge. The quartz region developed more pronounced and regular conical structures, with groove depths increasing to ~150 μm. These features reflected the enhanced energy deposition and constructive accumulation from repeated scanning, which promoted microstructure formation via self-organization mechanisms. In contrast, the chlorite region exhibited more irregular and less defined morphologies, accompanied by moderate depth increases (~130 μm). The chlorite’s layered silicate structure and lower thermal stability likely made it more susceptible to softening and partial melting rather than well-structured ablation.

At 6 passes (Figure 16c), the quartz surface achieved well-defined high-aspect-ratio conical structures with depths exceeding 190 μm. These microstructures were often decorated with secondary nanostructures, consistent with hierarchical texturing observed under SEM. Such features are favorable for surface functionality (e.g., hydrophobicity). The chlorite surface showed increased depth and roughness but lacked regularity. The features appeared partially melted and porous, indicating greater thermal damage and material ejection due to chlorite’s lower resistance to laser-induced stress. In addition, at 8 passes (Figure 16d), both materials experienced structural degradation. In the quartz region, the conical features enlarged further but exhibited signs of fracture and delamination, with groove depths reaching ~220 μm. This damage was likely due to excessive cumulative thermal and mechanical stress, consistent with microcrack formation observed in SEM. In the chlorite region, the surface became highly irregular, with features resembling melted craters and chaotic textures. The depth also increased (~230 μm), but the structural integrity was severely compromised. The laser-induced plasma shockwaves and thermal expansion likely led to interlayer separation and outgassing in the chlorite matrix.

**Figure 17:**
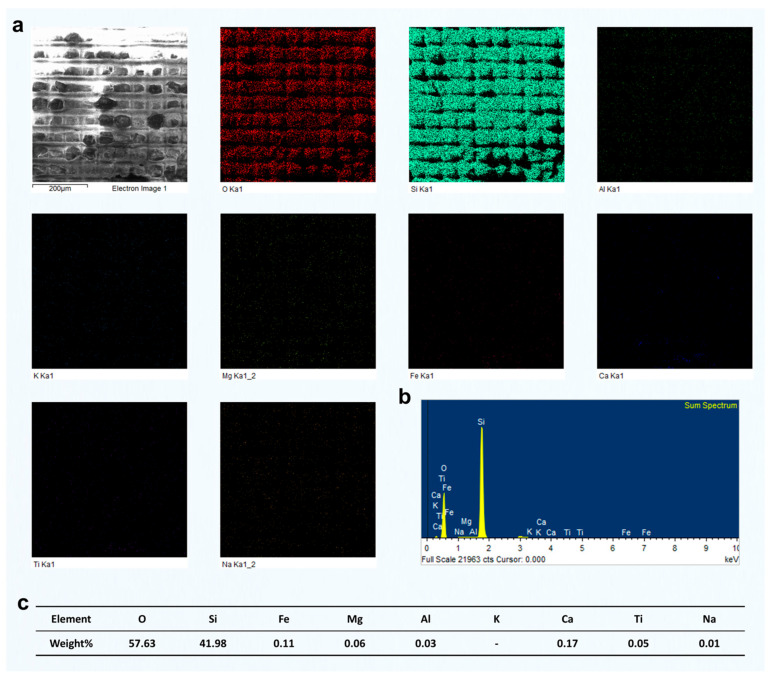
Elemental mapping and EDS analysis of the green schist with quartz as the major component after femtosecond laser grooving. A groove spacing of 60 μm was used and the structure was processed using two scans. (**a**) SEM image of the ablated area and corresponding elemental distribution maps (O, Si, Fe, Mg, Al, K, Ca, Ti, and Na). (**b**) EDS sum spectrum of the scanned region. (**c**) Elemental composition (weight %) of the selected area.

To further confirm the material composition and validate the grooving response observed under different scan passes, elemental mapping and EDS analysis were performed on the green schist sample after two femtosecond laser scans (Figure 17. Figure S6, in Supplementary S6, Supporting Information). The EDS mapping results in Figure 17a clearly revealed the dominant presence of Si and O, which corresponded well with the quartz-rich regions previously identified in the morphological and optical analyses (Figure 15 and Figure 16a). In contrast, other elements, such as Mg, Fe, Al, and Ca, were either sparsely distributed or nearly undetectable, indicating that the scanned area was primarily composed of quartz rather than chlorite. This compositional information corroborated the surface morphology features observed under low scanning passes. As shown in Figure 16a, the quartz region exhibited minimal topographic change, retaining a relatively flat surface with shallow rectangular grooves. The lack of significant structural transformation was attributed to the high ablation threshold and structural stability of quartz. The laser–material interaction under two-pass conditions, constrained by weak energy accumulation and limited pulse overlap, was insufficient to initiate notable restructuring or melting. The nearly pure Si signal peak in the EDS spectrum (Figure 17b), along with a high Si content of 41.98 wt% and O content of 57.63 wt% (Figure 17c), further supported the identification of quartz as the major constituent.

**Figure 18:**
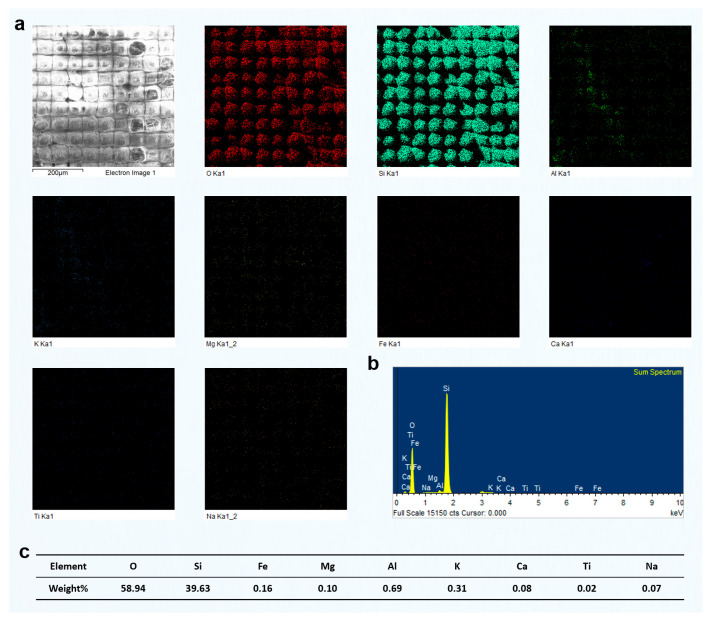
Elemental mapping and EDS analysis of the green schist with quartz as the major component after femtosecond laser grooving. A groove spacing of 60 μm was used and the structure was processed using four scans. (**a**) SEM image of the ablated area and corresponding elemental distribution maps (O, Si, Fe, Mg, Al, K, Ca, Ti, and Na). (**b**) EDS sum spectrum of the scanned region. (**c**) Elemental composition (weight %) of the selected area.

Figure 18 presents the elemental mapping and EDS results of the grooved green schist surface dominated by quartz, processed using four scanning passes at a fixed groove spacing of 60 μm. This dataset further deepens the insights established in Figure 15, Figure 16 and Figure 17 regarding the evolution of groove morphology and elemental redistribution during femtosecond laser processing. In Figure 18a, the SEM image and elemental maps confirm that the periodic grid pattern of grooves remains relatively well-defined after four scans. However, compared to the results after two scans (Figure 17a, Figure S7, in Supplementary S7, Supporting Information), the boundary between quartz-rich and chlorite-rich regions appears more blurred, indicating increased ablation and potential intermixing at the heterogeneous interface. Notably, the Si and O signals still dominate the elemental maps, consistent with the quartz-dominated composition, but there is a subtle increase in signal uniformity across the scanned area. This suggests partial ablation or re-deposition of materials, possibly from adjacent chlorite zones, due to the cumulative thermal and mechanical effects of multipass scanning.

The EDS spectrum in Figure 18b remains consistent with the quartz-rich nature of the region, showing a prominent Si peak alongside O. Minor signals from Mg, Al, K, and Ca are present, possibly reflecting either trace components of quartz or minimal contamination from chlorite, which becomes more exposed and susceptible to ablation as the groove depth increases. The compositional data in Figure 18c (58.94% O and 39.63% Si) align closely with Figure 17c, but the slight increase in Al (from 0.03% to 0.69%) and Mg (from 0.06% to 0.10%) further supports the notion of chlorite component exposure or diffusion after four passes. In conjunction with the 3D surface morphology observations in Figure 16b, the increased scanning pass resulted in visibly deeper and rougher grooves, particularly in quartz-rich zones. However, the surface roughness growth rate appears to taper, indicating that the ablation efficiency in quartz begins to saturate. Meanwhile, the chlorite-rich zones, with their lower ablation thresholds and greater susceptibility to multipulse modification, exhibit more disordered surface textures, supporting the idea of differential etching mechanisms.

### 3.5. Hydrophobic Performance of Green Schist After Femtosecond Laser Grooving

**Figure 19:**
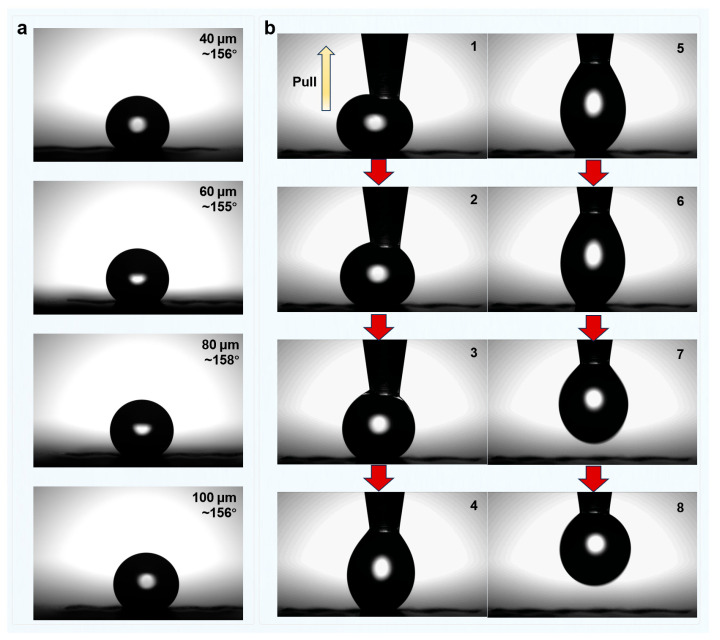
Hydrophobic performance test results of the green schist surface after femtosecond laser grooving. (**a**) Contact angles of surfaces grooved with different spacings under a single scanning pass. (**b**) Dynamic measurement of the hydrophobic behavior on the surface processed with two scanning passes at a 60 μm groove spacing, exhibiting superhydrophobic performance.

The wettability behavior of green schist surfaces after femtosecond laser processing was systematically examined using both static contact angle measurements and dynamic water adhesion tests (Figure 19 and Table 1). With a single scan and groove spacings of 40 μm and 60 μm, the laser-ablated surfaces exhibited contact angles approaching or exceeding 155°, reaching up to 158°. These results demonstrate that even under minimal processing conditions, the laser-induced micro/nanostructures were sufficient to confer significant hydrophobicity, with values that fall within or beyond the conventional threshold for superhydrophobicity (150°). To further assess adhesion behavior, dynamic water droplet tests were conducted under the condition of 60 μm spacing and two scan passes. As the needle was slowly lifted, the water droplet showed no sign of sticking to the surface; instead, it elongated and detached completely along with the needle. The hydrophobicity remained stable across all repetitions, indicating high reproducibility and process robustness. This clean lifting behavior is a typical manifestation of low adhesion or low contact angle hysteresis and is consistent with a superhydrophobic surface in the Cassie–Baxter wetting regime. Similar phenomena were also observed for surfaces processed with 4 and 6 scans, indicating that the induced wetting state was stable and robust over a range of processing doses.

The structural basis for this superhydrophobic behavior can be traced back to the microstructure evolution shown in Figure 15, Figure 16 and Figure 17. In quartz-dominated regions, the laser formed well-ordered arrays of micro-pits, which deepened with increasing scan number while preserving their periodic integrity. In contrast, the chlorite-rich domains exhibited slightly more disordered features due to differences in absorption and thermal properties, but up to 6 scans, the overall periodic structure remained intact. Elemental mapping and EDS analysis (Figure 18) further confirmed that Si and O remained the dominant elements, and the structural morphology was not accompanied by major compositional changes, underscoring the stability of the material under laser processing.

The excellent wettability performance aligns well with the Cassie–Baxter model, which describes a situation where liquid droplets are suspended on top of microstructured surfaces with air pockets trapped between asperities. This results in a composite solid–liquid–air interface, significantly reducing the effective contact area and promoting low adhesion and high contact angles. The combination of a high apparent contact angle and easy droplet detachment in our experiments is a direct reflection of this Cassie–Baxter state. A further analysis of scan number effects reveals that the Cassie–Baxter state can be reliably achieved with as few as two scans at 60 μm spacing. Additional passes (four and six scans) deepen the structures without compromising their continuity or functionality. However, as evidenced in Figure 16, excessive scanning (e.g., eight passes) may lead to partial structural collapse or merging, which can result in a wetting state transition toward the Wenzel regime—characterized by higher contact area and stronger droplet adhesion—ultimately compromising the uniformity and durability of the superhydrophobic response.

These findings highlight that femtosecond laser processing offers a controllable and effective method for engineering hierarchical structures on mineral surfaces. By fine-tuning the scanning parameters, stable Cassie–Baxter states with excellent water-repellent properties can be achieved on green schist substrates. This method provides a promising route for the functional modification of natural geological materials and expands the potential for their use in applications requiring tailored surface wettability and interfacial control. Considering the practical needs of stone artifact conservation protection, future studies should focus on the long-term stability and environmental durability of the laser-fabricated microstructures, in order to further advance their real-world applicability [44,45]. In addition, while the current experiments were performed on flat green schist samples under controlled laboratory conditions, practical application on cultural heritage stone surfaces will inevitably encounter additional challenges. In situ surfaces are often weathered, irregular, or oriented vertically, which may affect laser absorption and cleaning uniformity [1,9]. To address these issues, portable femtosecond laser systems with fiber delivery and flexible scanning heads offer promising adaptability for complex geometries. Furthermore, adaptive optimization of laser parameters will be necessary to account for variations in surface roughness and weathering. Future work should focus on validating the efficacy and safety of the cleaning process under real-world conservation conditions to ensure preservation without substrate damage.

## 4. Conclusions

Inspired by the superhydrophobic micro/nanostructures found on lotus leaves, the laser ablation technique for fabricating similar micro/nanostructures on stone surfaces has become a mature technology. However, considering the practical issue of stone artifact conservation, such as the green schist commonly used in the Wudang Mountain Rock Temple, which contains various heterogeneous components, like quartz, chlorite, muscovite, etc., their absorption of laser and response to processing differ significantly, posing a great challenge to the creation of uniform micro/nanostructures or stable superhydrophobic structures. Therefore, in this study, we systematically investigated the machining characteristics of green schist's different components under different grooving parameters, including surface morphology, three-dimensional structure, and composition changes, and confirmed the superhydrophobic performance of the fabricated micro/nanostructures. The main findings are as follows:

(1) The texture of green schist is significantly different between its transverse and longitudinal sections. The transverse texture has a more uniform composition, making it more suitable for micro/nanostructure fabrication. The composition consists of quartz (~40%), chlorite (~35%), muscovite (~15%), biotite (~4%), carbonates (~4%), and minor dust-like carbonaceous matter (~2%). SEM results from selected samples show that certain areas contain detectable metallic elements, such as Fe and Ni.

(2) In addition, analysis of surface morphology, three-dimensional structure, and element distribution under femtosecond laser grooving revealed notable differences among quartz, chlorite, and muscovite. Quartz, with the lowest laser absorption due to minimal nonlinear effects, produced the most stable surface, making it the most resilient of the three components. Chlorite, with high Fe and Mg content, absorbs laser primarily in a linear manner, resulting in the greatest material removal under the same parameters, making it prone to excessive ablation. Muscovite exhibits typical thermal effects under femtosecond laser irradiation, showing heating, melting, and expansion.

(3) Finally, we systematically investigated the effects of different groove spacings (100 μm, 80 μm, 60 μm, and 40 μm) and scan passes on the various components of green schist. We identified optimal processing parameters (60 μm groove spacing, 2–6 scan passes) that achieved stable superhydrophobic performance. Specifically, under these parameters, the quartz component in green schist formed a stable conical structure, closely resembling the superhydrophobic structure of lotus leaves.

Theoretically, it provides new insights into the behavior of heterogeneous stones, like green schist, during femtosecond laser grooving, enriching stone laser processing research. Practically, the optimized laser parameters enhance the superhydrophobic performance of stone, improving durability and reducing erosion from water and pollutants, thereby extending the lifespan of artifacts. Thus, this study offers new ideas and techniques for the conservation of stone artifacts, especially those with complex heterogeneous compositions. In the future, analyzing the response differences of various mineral components to femtosecond laser irradiation using the two-temperature model [46] would be a meaningful approach to guide and potentially predict the processing performance.

## Figures and Tables

**Table 1:** Relationship between process parameters and the water contact angle.

Groove Spacing μm	Scanning Passes	Water Contact Angle °
40	1	156
60	1	155
80	1	158
100	1	156
40	2	156
60	2	Superhydrophobic surface
80	2	167
100	2	158

## Data Availability

The data that support the findings of this study are available from the authors upon reasonable request.

## Supplementary Materials

The following supporting information can be downloaded at: https://www.mdpi.com/article/10.3390/ma18163751/s1, Figure S1: Optical microscope analysis of the composition of axial green schist under plane polarized light revealed notable differences between the two samples. (a) and (b) are two different samples; Figure S2: Comparison of greenschist surface structures processed by femtosecond laser grooving with different groove spacings. The structure was processed using four scans. (a) A groove spacing of 100 μm. (b) A groove spacing of 80 μm. (c) A groove spacing of 60 μm. (d) A groove spacing of 40 μm. (i) shows the overall morphology of the femtosecond laser-grooved region, (ii,iii) show the surface morphology of the quartz-rich region after femtosecond laser grooving, (iv) shows the surface morphology of the chlorite or muscovite-rich region after femtosecond laser grooving; Figure S3: Comparison of greenschist surface structures processed by femtosecond laser grooving with different groove spacings. The structure was processed using six scans. (a) A groove spacing of 100 μm. (b) A groove spacing of 80 μm. (c) A groove spacing of 60 μm. (d) A groove spacing of 40 μm. (i) shows the overall morphology of the femtosecond laser-grooved region, (ii,iii) show the surface morphology of the quartz-rich region after femtosecond laser grooving, (iv) shows the surface morphology of the chlorite or muscovite-rich region after femtosecond laser grooving; Figure S4: Comparison of greenschist surface structures processed by femtosecond laser grooving with different groove spacings. The structure was processed using eight scans. (a) A groove spacing of 100 μm. (b) A groove spacing of 80 μm. (c) A groove spacing of 60 μm. (d) A groove spacing of 40 μm. (i) shows the overall morphology of the femtosecond laser-grooved region, (ii,iii) show the surface morphology of the quartz-rich region after femtosecond laser grooving, (iv) shows the surface morphology of the chlorite or muscovite-rich region after femtosecond laser grooving; Figure S5: Comparison of greenschist surface structures processed by femtosecond laser grooving with different groove spacings. The structure was processed using ten scans. (a) A groove spacing of 100 μm. (b) A groove spacing of 80 μm. (c) A groove spacing of 60 μm. (d) A groove spacing of 40 μm. (i) shows the overall morphology of the femtosecond laser-grooved region, (ii,iii) show the surface morphology of the quartz-rich region after femtosecond laser grooving, (iv) shows the surface morphology of the chlorite or muscovite-rich region after femtosecond laser grooving; Figure S6: Elemental mapping and EDS analysis of the green schist with chlorite as the major component after femtosecond laser grooving. A groove spacing of 60 μm was used and the structure was processed using two scans. (a) SEM image of the ablated area and corresponding elemental distribution maps (O, Si, Fe, Mg, Al, K, Ca, Ti and Na). (b) EDS sum spectrum of the scanned region. (c) Elemental composition (weight %) of the selected area; Figure S7: Elemental mapping and EDS analysis of the green schist with muscovite as the major component after femtosecond laser grooving. A groove spacing of 60 μm was used and the structure 1119 was processed using four scans. (a) SEM image of the ablated area and corresponding elemental distribution maps (O, Si, Fe, Mg, Al, K, Ca, Ti and Na). (b) EDS sum spectrum of the scanned region. (c) Elemental composition (weight %) of the selected area.

## References

[1] Wang C., Chen M., Wang Y. (2023). Surface flaking mechanism of stone components of ancient building complex in Wudang Mountain, China. Constr. Build. Mater..

[2] Wu X. (2015). Wild Edible Plants and Pilgrimage on Wudang Mountain. J. Ethnobiol..

[3] Lei Z., Wan L., Zhang Y. (2018). Investigation, Diagnosis, Assessment and Conservation Strategy for a Wall Painting at Wudang Mountain Taoist Temple Using BIM Technology. Stud. Conserv..

[4] Sadat-Shojai M., Ershad-Langroudi A. (2009). Polymeric coatings for protection of historic monuments: Opportunities and challenges. J. Appl. Polym. Sci..

[5] Wang N., Wang Q., Xu S., Lei L. (2021). Green fabrication of mechanically stable superhydrophobic concrete with anti–corrosion property. J. Clean. Prod..

[6] Xu S., Wang Q., Wang N., Qu L., Song Q. (2021). Study of corrosion property and mechanical strength of eco–friendly fabricated superhydrophobic concrete. J. Clean. Prod..

[7] Schnell G., Polley C., Thomas R., Bartling S., Wagner J., Springer A., Seitz H. (2022). How droplets move on laser–structured surfaces: Determination of droplet adhesion forces on nano– and microstructured surfaces. J. Colloid Interface Sci..

[8] Eryildiz B., Ozbey-Unal B., Menceloglu Y.Z., Keskinler B., Koyuncu I. (2023). Development of robust superhydrophobic PFA/TMI/PVDF membrane by electrospinning/electrospraying techniques for air gap membrane distillation. J. Appl. Polym. Sci..

[9] Wang C., Zhang Y., Hu X., Jia X., Li K., Wang C., Wang Y. (2024). Sustainability–oriented construction materials for traditional residential buildings: From material characteristics to environmental suitability. Case Stud. Constr. Mater..

[10] Bai X., Yang S., Tan C., Jia T., Guo L., Song W., Jian M., Zhang X., Zhang Z., Wu L. (2022). Synthesis of TiO_2_ based superhydrophobic coatings for efficient anti–corrosion and self–cleaning on stone building surface. J. Clean. Prod..

[11] López A., Pozo–Antonio J., Moreno A., Rivas T., Pereira D., Ramil A. (2022). Femtosecond laser texturing as a tool to increase the hydrophobicity of ornamental stone: The influence of lithology and texture. J. Build. Eng..

[12] Díaz A.L., Ramil A., Freire-Lista D.M. (2023). Evaluation of femtosecond laser texturing on carbonate heritage stones. Lasers in the Conservation of Artworks XIII2023.

[13] Nosonovsky M., Bhushan B. (2008). Biologically Inspired Surfaces: Broadening the Scope of Roughness. Adv. Funct. Mater..

[14] Darmanin T., Guittard F. (2015). Superhydrophobic and superoleophobic properties in nature. Mater. Today.

[15] Zhao J., Gao X., Chen S., Lin H., Li Z., Lin X. (2022). Hydrophobic or superhydrophobic modification of cement–based materials: A systematic review. Compos. Part B Eng..

[16] Ariza R., Alvarez-Alegria M., Costas G., Tribaldo L., Gonzalez-Elipe A.R., Siegel J., Solis J. (2022). Multiscale ultrafast laser texturing of marble for reduced surface wetting. Appl. Surf. Sci..

[17] Zhang C., Zhang X., Shen H., Shuai D., Xiong X., Wang Y., Huang H., Li Y. (2023). Superior self–cleaning surfaces via the synergy of superhydrophobicity and photocatalytic activity: Principles, synthesis, properties, and applications. J. Clean. Prod..

[18] Wang J., Zhang Y., He Q. (2022). Durable and robust superhydrophobic fluororubber surface fabricated by template method with exceptional thermostability and mechanical stability. Sep. Purif. Technol..

[19] Carrascosa L.A., Zarzuela R., Botana-Galvín M., Botana F.J., Mosquera M.J. (2022). Achieving superhydrophobic surfaces with tunable roughness on building materials via nanosecond laser texturing of silane/siloxane coatings. J. Build. Eng..

[20] Zhao D., Zhu H., Zhang Z., Xu K., Lei W., Gao J., Liu Y. (2022). Transparent superhydrophobic glass prepared by laser–induced plasma–assisted ablation on the surface. J. Mater. Sci..

[21] Guo C., Li K., Liu Z.L., Chen Y., Xu J., Li Z., Cui W., Song C., Wang C., Jia X. (2025). CW laser damage of ceramics induced by air filament. Opto-Electron. Adv..

[22] Jia X., Luo J., Li K., Wang C., Li Z., Wang M., Jiang Z., Veiko V.P., Duan J. (2025). Ultrafast laser welding of transparent materials: From principles to applications. Int. J. Extreme Manuf..

[23] Zhang J., Hu Y., Wang F., Liu Q., Niu F., Li J., Huang M., Zhang G., Sun R. (2024). Precise modulation of the debonding behaviours of ultra–thin wafers by laser–induced hot stamping effect and thermoelastic stress wave for advanced packaging of chips. Int. J. Extreme Manuf..

[24] Liu C., Zheng J., Liu X., Yin K., Wang H., Wang Q. (2023). Facile laser–based process of superwetting zirconia ceramic with adjustable adhesion for self–cleaning and lossless droplet transfer. Appl. Surf. Sci..

[25] Zhang B., Wang Z., Tan D., Gu M., Yue Y., Qiu J. (2024). Focal volume optics for composite structuring in transparent solids. Int. J. Extreme Manuf..

[26] Wan H., Shu Y., Chen S., Cao H., Zhou S., Liu S., Gui C. (2024). Laser–induced thermo–compression bonding for Cu–Au heterogeneous nanojoining. Int. J. Extreme Manuf..

[27] Chantada A., Penide J., Riveiro A., del Val J., Quintero F., Meixus M., Soto R., Lusquiños F., Pou J. (2017). Increasing the hydrophobicity degree of stonework by means of laser surface texturing: An application on Zimbabwe black granites. Appl. Surf. Sci..

[28] Tian Z., Lei Z., Chen X., Chen Y., Zhang L.-C., Bi J., Liang J. (2020). Nanosecond pulsed fiber laser cleaning of natural marine micro–biofoulings from the surface of aluminum alloy. J. Clean. Prod..

[29] Pou–Álvarez P., Riveiro A., Nóvoa X., Fernández-Arias M., del Val J., Comesaña R., Boutinguiza M., Lusquiños F., Pou J. (2021). Nanosecond, picosecond and femtosecond laser surface treatment of magnesium alloy: Role of pulse length. Surf. Coat. Technol..

[30] López A.J., Ramil A., Pozo-Antonio J.S., Rivas T., Pereira D. (2019). Ultrafast Laser Surface Texturing: A Sustainable Tool to Modify Wettability Properties of Marble. Sustainability.

[31] Sun X., Wang K., Fan Z., Wang R., Mei X., Lu Y. (2021). Regulation of hydrophobicity on yttria stabilized zirconia surface by femtosecond laser. Ceram. Int..

[32] Wang Q., Kainuma S., Zhuang S., Shimizu K., Haraguchi M. (2022). Laser cleaning on severely corroded steel members: Engineering attempt and cleanliness assessment. J. Clean. Prod..

[33] Xu J., Su Q., Shan D., Guo B. (2019). Sustainable micro–manufacturing of superhydrophobic surface on ultrafine–grained pure aluminum substrate combining micro–embossing and surface modification. J. Clean. Prod..

[34] Balage P., Lopez J., Bonamis G., Hönninger C., Manek-Hönninger I. (2022). Crack–free high–aspect ratio holes in glasses by top–down percussion drilling with infrared femtosecond laser GHz–bursts. Int. J. Extreme Manuf..

[35] Orazi L., Romoli L., Schmidt M., Li L. (2021). Ultrafast laser manufacturing: From physics to industrial applications. CIRP Ann..

[36] Tan D., Zhang B., Qiu J. (2021). Ultrafast Laser Direct Writing in Glass: Thermal Accumulation Engineering and Applications. Laser Photon. Rev..

[37] Ding Y., Liu L., Wang C., Li C., Lin N., Niu S., Han Z., Duan J. (2023). Bioinspired Near–Full Transmittance MgF_2_ Window for Infrared Detection in Extremely Complex Environments. ACS Appl. Mater. Interfaces.

[38] Yang Z., Tian C., Ren H., Wei X., Shen H. (2024). Welding threshold in ultrafast laser welding of quartz glass and 304 stainless steel. Opt. Laser Technol..

[39] Shugaev M.V., Wu C., Armbruster O., Naghilou A., Brouwer N., Ivanov D.S., Derrien T.J.-Y., Bulgakova N.M., Kautek W., Rethfeld B. (2016). Fundamentals of ultrafast laser–material interaction. MRS Bull..

[40] Qingliang S., Tiyuan W., Qiang S., Fang Y., Hejun L., Fu M. (2022). Unraveling of the laser drilling of carbon/carbon composites: Ablation mechanisms, shape evolution, and damage evaluation. Int. J. Mach. Tools Manuf..

[41] Shen H., Yang Z., Tian C., Ren H., Wei X. (2025). High welding strength of fused silica and stainless steel by picosecond laser with large defocus. Ceram. Int..

[42] Fan P., Dong X., Wang K., Liu B., Shen P., Yi L., Mei X., Fan Z. (2024). Optimization of laidback fan–shaped holes machined by femtosecond laser. Int. J. Mech. Sci..

[43] Jia X., Lin J., Li Z., Wang C., Li K., Wang C., Duan J. (2025). Continuous wave laser ablation of alumina ceramics under long focusing condition. J. Manuf. Process..

[44] Zolriasatein A., RajabiMashhadi Z., Rezaei Abadchi M., Riahi Noori N., Abyazi S. (2022). A New Approach Based on RTV/SiO_2_ Nano coating to Tackling Environmental Pollution on Electrical Energy Distributions. J. Renew. Energy Environ..

[45] Zolriasatein A., RajabiMashhadi Z., Ardebili D.H., Noori N.R., Abadchi M.R., Mirzaee M. (2023). UV accelerated aging of RTV/SiO_2_ nanocomposites: Study on surface microstructure, hydrophobicity, and electrical properties. Int. J. Adhes. Adhes..

[46] Li Z., Lin J., Wang C., Li K., Jia X., Wang C., Duan J. (2024). Damage performance of alumina ceramic by femtosecond laser induced air filamentation. Opt. Laser Technol..

