# Supplementary material for: Surface Microstructural Responses of Heterogeneous Green Schist to Femtosecond Laser Grooving with Varying Process Parameters
Source: Materials (Basel). 2025 Aug 11;18(16):3751. doi: 10.3390/ma18163751 (PMC12387500; doi:10.3390/ma18163751)
Supplement: Supplementary file 1 [file materials-18-03751-s001.zip › materials-3661926-supplementary.docx]

Supporting Information

Surface Microstructural Responses of Heterogeneous Green Schist to Femtosecond Laser Grooving with Varying Process Parameters

Chengaonan Wang ^1^ Kai Li ^2^, Xianshi Jia ^2^, Cong Wang ^2^, Yansong Wang ^1^ and Zheng Yuan ^1,^*

^1^School of Urban Design, Wuhan University, Hubei 430072, China

^2^State Key Laboratory of Precision Manufacturing for Extreme Service Performance, College of Mechanical and Electrical Engineering, Central South University, Changsha 410083, China

***** Correspondence: 00011299@whu.edu.cn


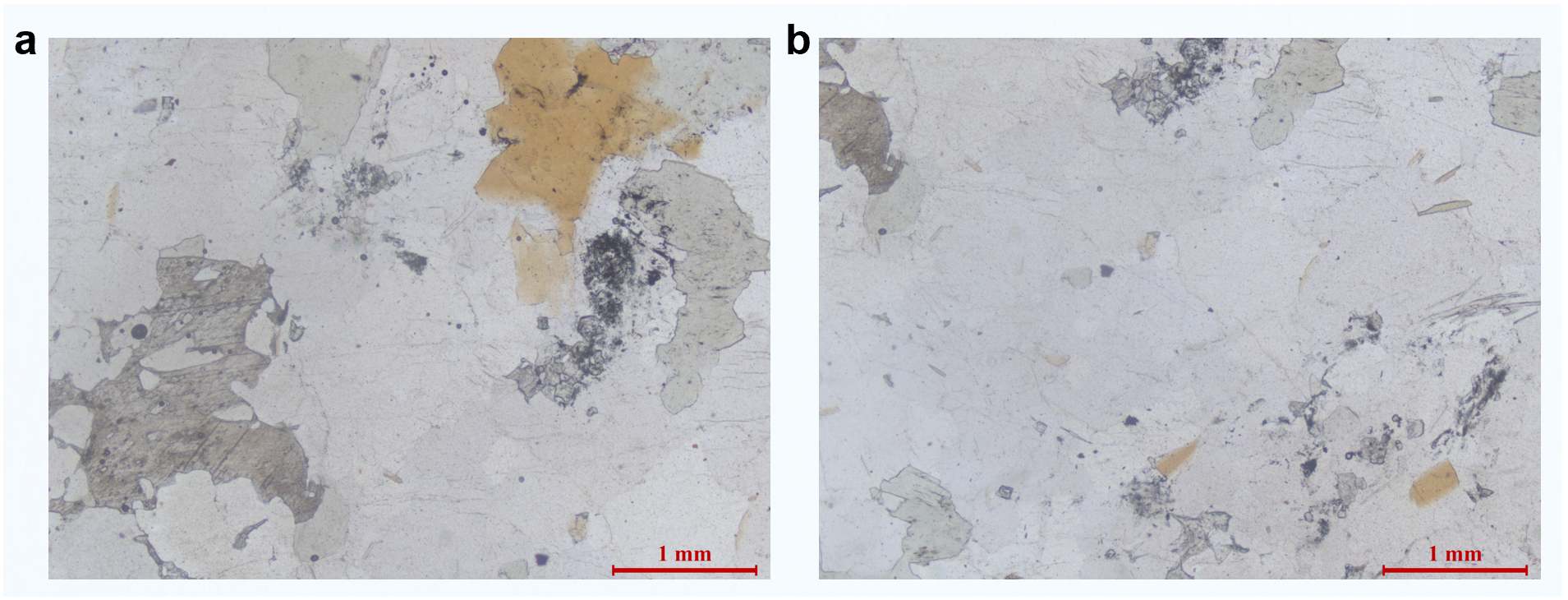


**Figure S1.** Optical microscope analysis of the composition of axial green schist under plane polarized light revealed notable differences between the two samples. (a) and (b) are two different samples.


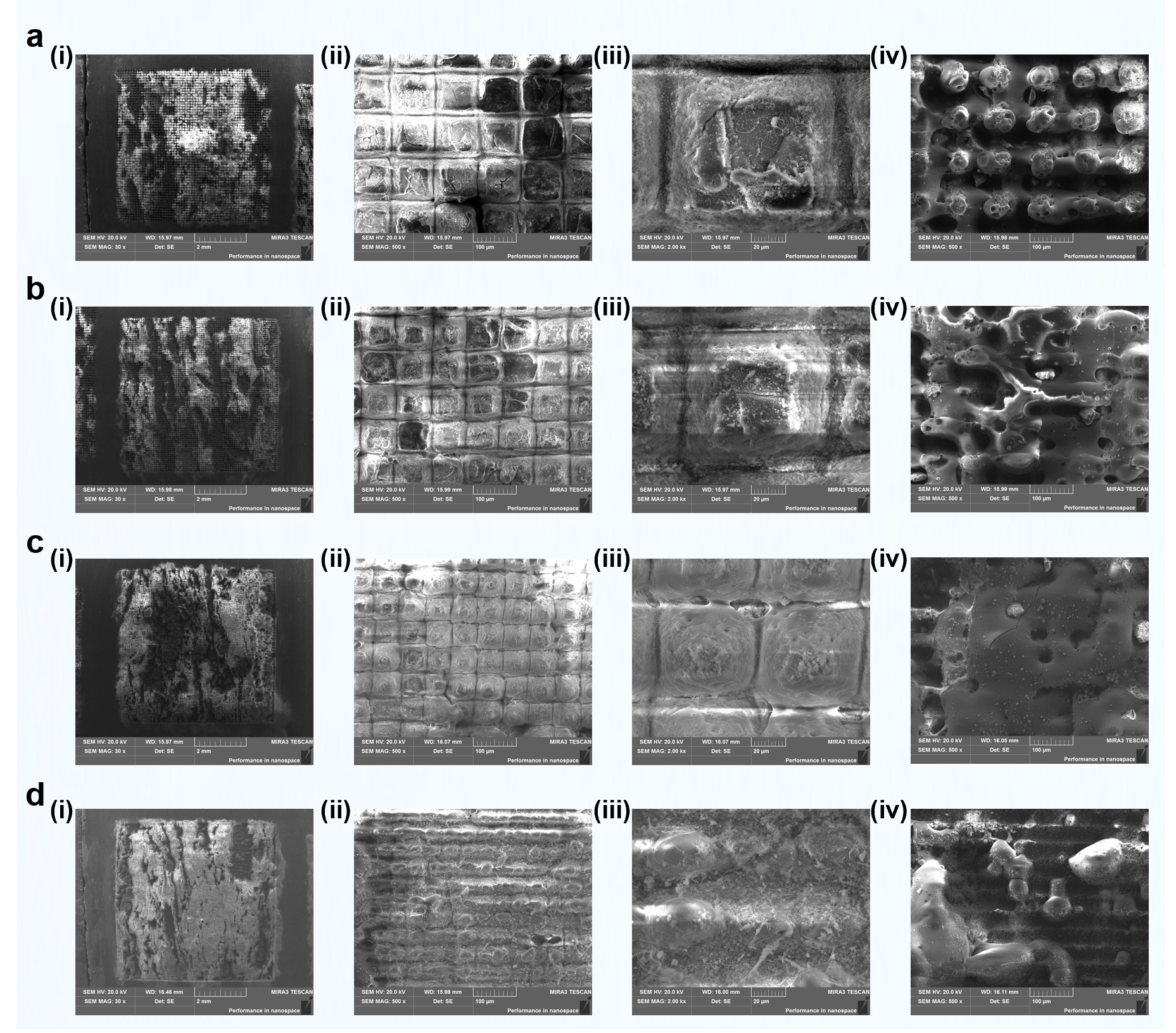


**Figure S2.** Comparison of greenschist surface structures processed by femtosecond laser grooving with different groove spacings. The structure was processed using four scans. (a) A groove spacing of 100 μm. (b) A groove spacing of 80 μm. (c) A groove spacing of 60 μm. (d) A groove spacing of 40 μm. (i) shows the overall morphology of the femtosecond laser-grooved region, (ii-iii) show the surface morphology of the quartz-rich region after femtosecond laser grooving, (iv) shows the sur-face morphology of the chlorite or muscovite-rich region after femtosecond laser grooving.


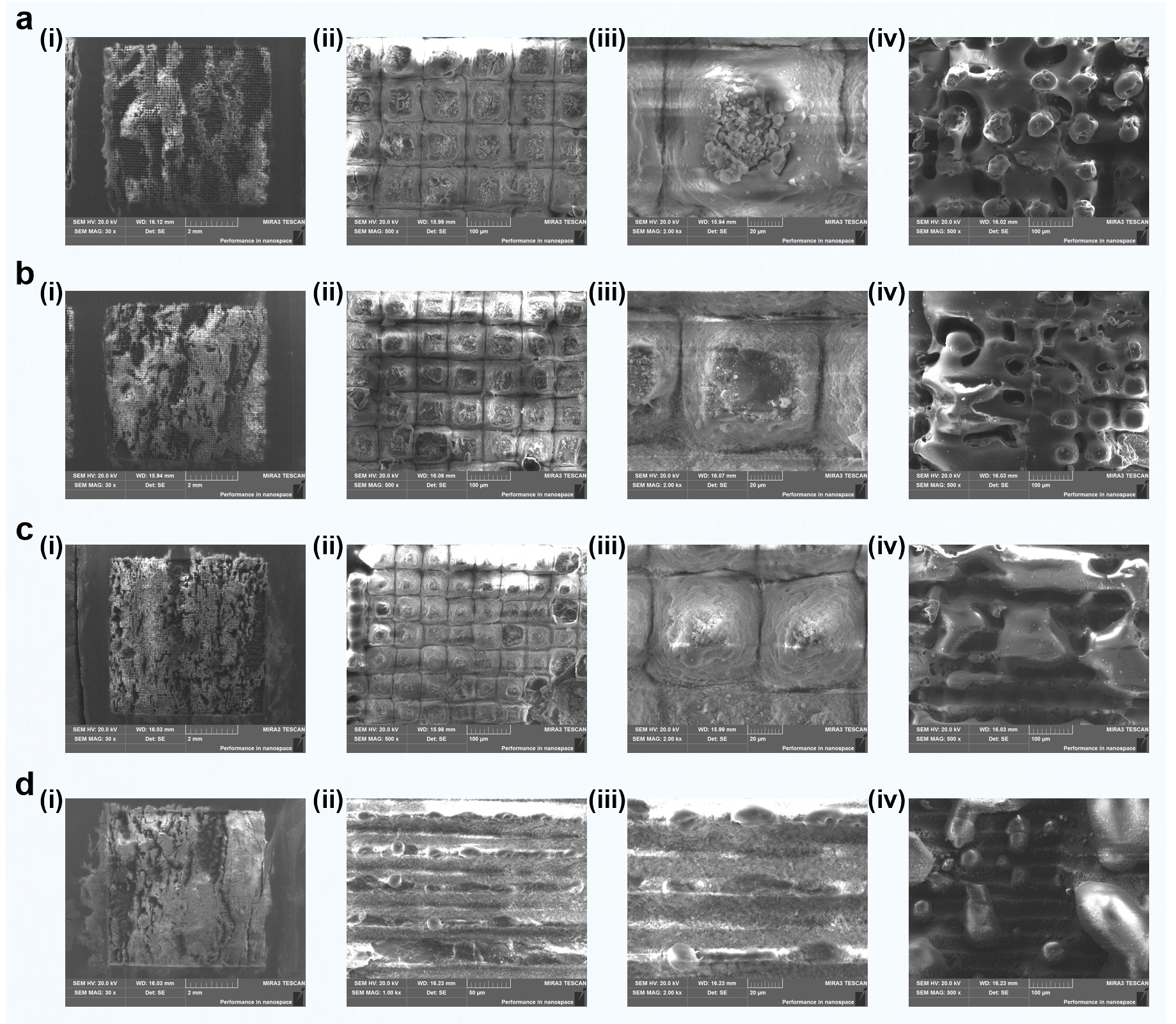


**Figure S3.** Comparison of greenschist surface structures processed by femtosecond laser grooving with different groove spacings. The structure was processed using six scans. (a) A groove spacing of 100 μm. (b) A groove spacing of 80 μm. (c) A groove spacing of 60 μm. (d) A groove spacing of 40 μm. (i) shows the overall morphology of the femtosecond laser-grooved region, (ii-iii) show the surface morphology of the quartz-rich region after femtosecond laser grooving, (iv) shows the sur-face morphology of the chlorite or muscovite-rich region after femtosecond laser grooving.


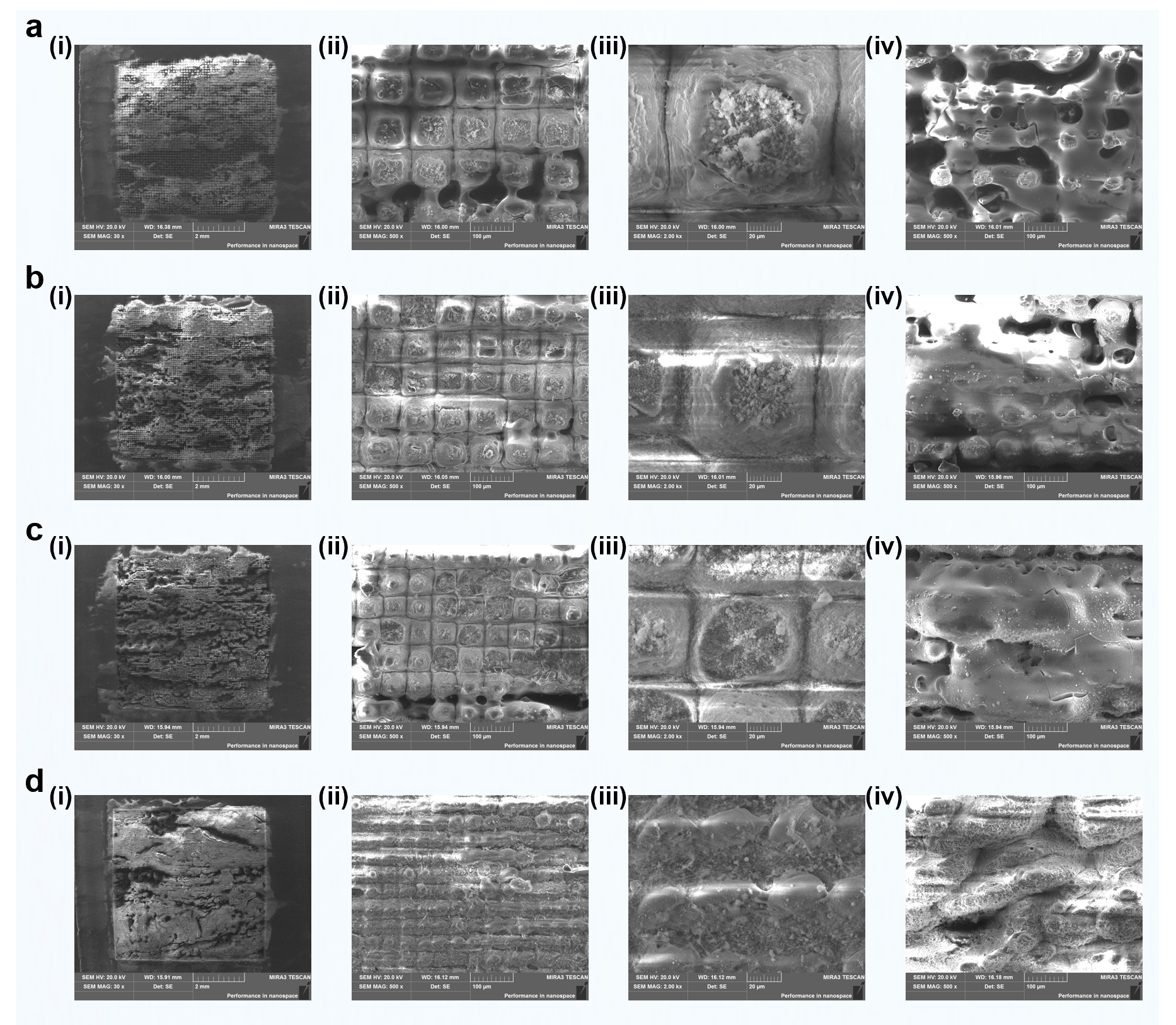


**Figure S4.** Comparison of greenschist surface structures processed by femtosecond laser grooving with different groove spacings. The structure was processed using eight scans. (a) A groove spacing of 100 μm. (b) A groove spacing of 80 μm. (c) A groove spacing of 60 μm. (d) A groove spacing of 40 μm. (i) shows the overall morphology of the femtosecond laser-grooved region, (ii-iii) show the surface morphology of the quartz-rich region after femtosecond laser grooving, (iv) shows the sur-face morphology of the chlorite or muscovite-rich region after femtosecond laser grooving.


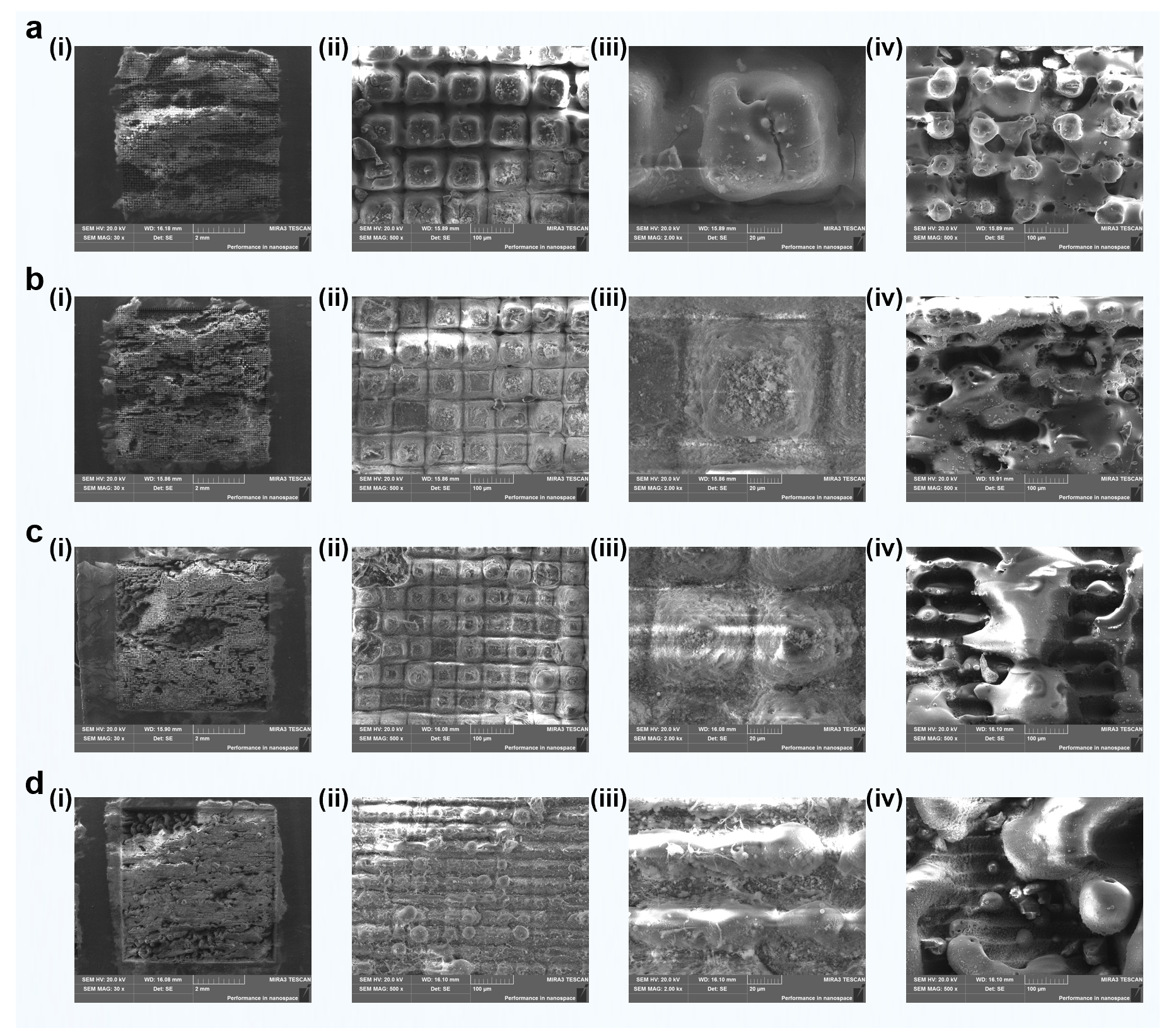


**Figure S5.** Comparison of greenschist surface structures processed by femtosecond laser grooving with different groove spacings. The structure was processed using ten scans. (a) A groove spacing of 100 μm. (b) A groove spacing of 80 μm. (c) A groove spacing of 60 μm. (d) A groove spacing of 40 μm. (i) shows the overall morphology of the femtosecond laser-grooved region, (ii-iii) show the surface morphology of the quartz-rich region after femtosecond laser grooving, (iv) shows the sur-face morphology of the chlorite or muscovite-rich region after femtosecond laser grooving.


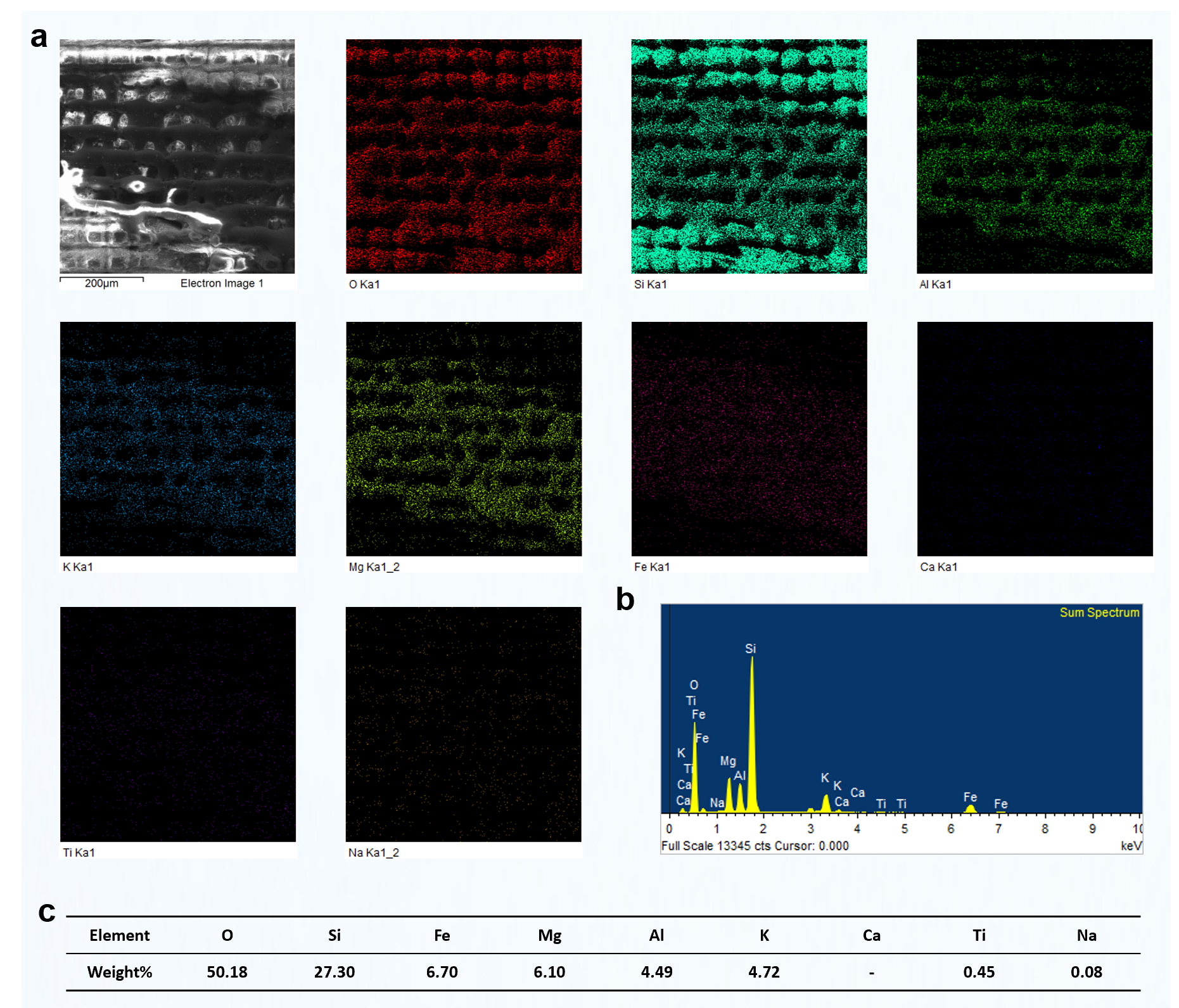


**Figure S6.** Elemental mapping and EDS analysis of the green schist with chlorite as the major component after femtosecond laser grooving. A groove spacing of 60 μm was used and the structure was processed using two scans. (a) SEM image of the ablated area and corresponding elemental distribution maps (O, Si, Fe, Mg, Al, K, Ca, Ti and Na). (b) EDS sum spectrum of the scanned region. (c) Elemental composition (weight %) of the selected area.


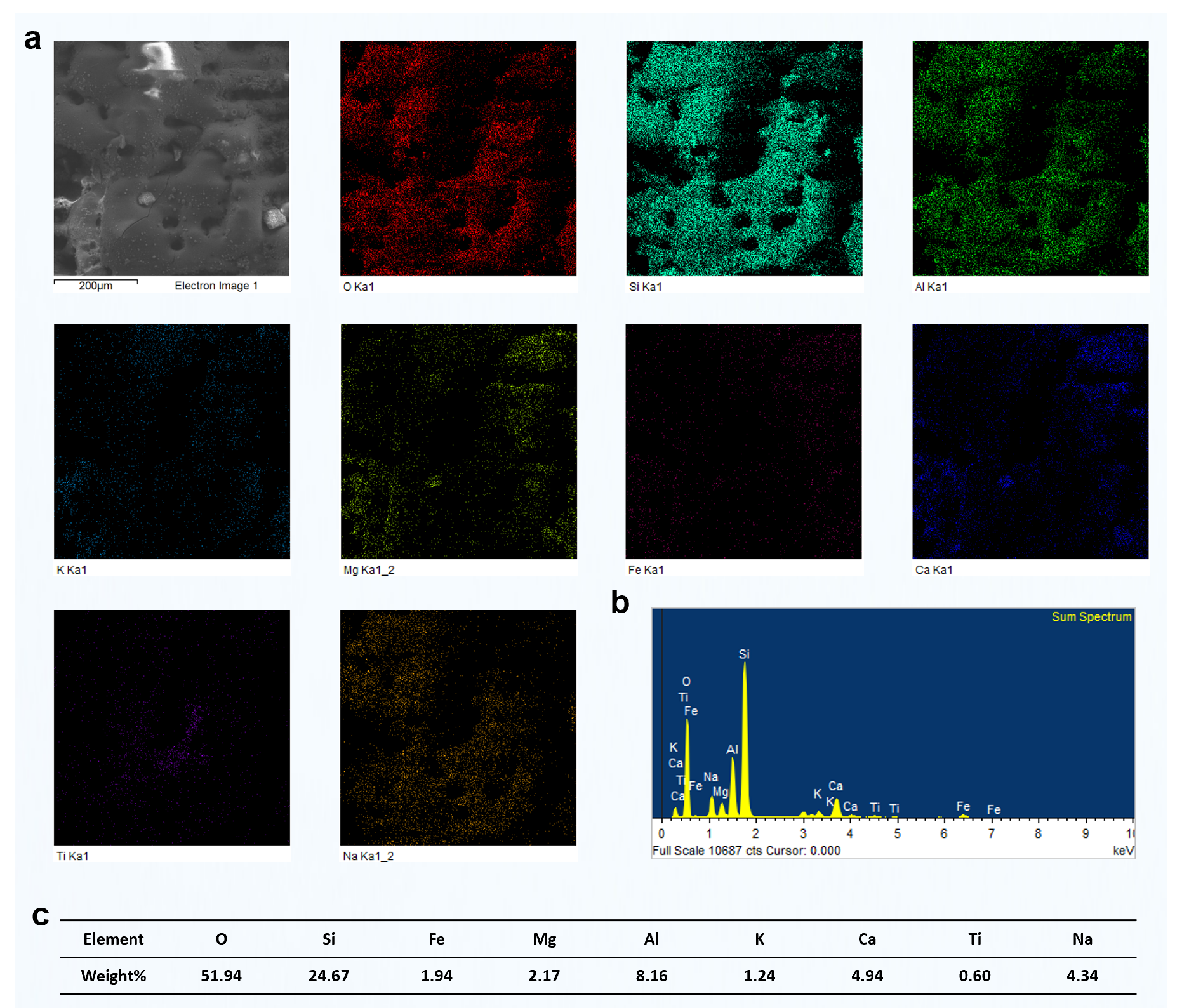


**Figure S7.** Elemental mapping and EDS analysis of the green schist with muscovite as the major component after femtosecond laser grooving. A groove spacing of 60 μm was used and the structure 1119 was processed using four scans. (a) SEM image of the ablated area and corresponding elemental distribution maps (O, Si, Fe, Mg, Al, K, Ca, Ti and Na). (b) EDS sum spectrum of the scanned region. (c) Elemental composition (weight %) of the selected area.
